# Whether radiofrequency thermocoagulation guided by stereotactic electroencephalography can benefit drug-resistant epilepsy in the early follow-up stage

**DOI:** 10.1186/s42494-025-00207-5

**Published:** 2025-03-05

**Authors:** Jingtao Yan, Yuhao Wang, Le Wang, Weipeng Jin, Chuan Du, Guangfeng Li, Deqiu Cui, Shaoya Yin

**Affiliations:** 1 Clinical College of Neurology, Neurosurgery and Neurorehabilitation, Tianjin Medical University, Tianjin, 300350 China; 2 Department of Neurosurgery, Jin Cheng People’s Hospital, Jincheng City, Shanxi Province 048026 China; 3 Department of Neurosurgery, Huanhu Hospital, Tianjin University, Tianjin, 300350 China

**Keywords:** RF-TC guided by SEEG, Drug resistant epilepsy, Engel grading

## Abstract

**Background:**

Stereotactic electroencephalography (SEEG) has emerged as a widely utilized diagnostic approach in epilepsy surgery, demonstrating broad clinical applications and a favorable safety profile. SEEG, when combined with radiofrequency thermocoagulation (RF-TC), facilitates the identification of epileptogenic zones and serves as a therapeutic option that eliminates the need for general anesthesia, thus incuring no additional costs for patients. This study aimed to investigate whether SEEG-guided RF-TC provides early therapeutic benefits.

**Methods:**

A retrospective analysis was performed on 44 patients with drug-resistant epilepsy who underwent RF-TC treatment between April 2019 and December 2022, with complete follow-up data available. RF-TC was administered after the recording three or more habitual epileptic seizures in all patients. Demographic characteristics were retrospectively assessed, and treatment outcomes were evaluated using the Engel classification system.

**Results:**

SEEG-guided RF-TC treatment was successfully performed in all patients without significant neurological complications. An average of 7.7 ± 0.4 electrodes were implanted per patient, with a SEEG monitoring duration of 7.5 days (range: 6.8–11). Follow-up after thermocoagulation ranged from 9 to 63 months. At the three-month follow-up, 56.8% of patients achieved Engel I (11 cases) and II (14 cases) were included. At the six-month follow-up, 40.9% of patients achieved Engel grades I (9 cases) and II (9 cases), with five patients proceeding to surgical treatment. By the 12-month follow-up, 40.9% of patients reached Engel grades I (5 cases) and II (13 cases), with a cumulative total of 12 patients undergoing surgical intervention. At the 24-month follow-up, 20.5% of patients achieved Engel grades I (3 cases) and II (6 cases), resulting in a cumulative total of 16 patients undergoing surgical treatment. A statistically significant reduction in seizure frequency was observed before and after thermocoagulation in all 44 patients (*P* = 0.007), although the therapeutic effect of thermocoagulation decreased over time.

**Conclusions:**

SEEG-guided RF-TC is a safe and effective treatment modality for drug-resistant epilepsy. However, as follow-up duration increases, both seizure-free rates and response rates following RF-TC progressively decline.

**Supplementary Information:**

The online version contains supplementary material available at 10.1186/s42494-025-00207-5.

## Background

Drug-resistant epilepsy (DRE) is defined as the persistence of seizures despite the use of two appropriately chosen and tolerable antiseizure medication regimens, either as monotherapy or in combination. Some patients with DRE require preoperative evaluation and surgical intervention. Stereotactic electroencephalography (SEEG), a minimally invasive and safe diagnostic and therapeutic approach, allows the implantation of multiple electrodes into specific targets within the skull using stereotactic techniques. This method facilitates the precise identification of the location, extent, and epileptic network of the seizure focus [1–3]. Furthermore, SEEG-guided radiofrequency thermocoagulation (RF-TC) can ablate the epileptic focus and associated network by utilizing specific electrode contacts, thereby achieving therapeutic goals.

Since the first application of SEEG-guided RF-TC in 2004, numerous studies have demonstrated its safety and efficacy. However, the reported duration of its therapeutic effects and postoperative seizure-free rates vary significantly across different centers [4–6].

Clinical data and follow-up results from 44 patients with DRE who underwent SEEG-guided RF-TC in the Department of Neurosurgery at Tianjin Huanhu Hospital between April 2018 and December 2022 were collected for analysis. Through a retrospective review of these clinical data, this study aimed to evaluate the therapeutic effects of SEEG-guided RF-TC on DRE and explore its potential benefits in the early stages of treatment.

## Methods

### Patients

Over the past five years, SEEG-guided RF-TC has been performed on 52 patients with DRE, of whom 44 met the criteria for inclusion in this retrospective study. The inclusion criteria [7] were as follows: (1) medically refractory epilepsy, defined as seizures that could not be adequately controlled with two antiseizure medication regimens, failing to reduce seizure frequency by at least 50%; (2) inability to precisely localize epileptic foci through preoperative first-stage evaluations or cases where epileptic foci were adjacent to functional areas, necessitating SEEG implantation to test hypotheses regarding the epileptic focus; and (3) approval for the procedure by the Ethics Committee of Tianjin Huanhu Hospital, with informed consent obtained from patients and their families. 

Demographic and clinical data collected for analysis included age, gender, duration of epilepsy, seizure frequency, medical history, epilepsy etiology, number of previously used antiseizure medications, seizure frequency before and after RF-TC, subsequent surgical intervention, and Engel grading. Detailed information is presented in Table 1.

**Table 1:** Clinical characteristics of the study population undergoing RF-TC guided by SEEG procedures (*n* = 44)

Characteristics	Number(*n)*	Range	Median (IQR) or Mean ± SD
Age at seizure onset (years)	44	13–50	28.6 (22.0–37.5)
Sex (male/female)	31/13	-	-
Duration of epileptic seizures (years)		0.1–39	6.5 (0.8, 11.3)
Seizure frequency (D/W/M)	10/13/21	-	-
Number of ASMs administration before RF-TC (n)	-	1–4	2 (1, 3)
Etiology of epilepsy			
Malformations of cortical development	4	-	-
Polymicrogyria	1	-	-
Focal cortical dysplasia	3	-	-
Neurodevelopmental tumors	3	-	-
Hippocampal sclerosis	12	-	-
Cranial trauma	2	-	-
Cerebrovascular disease	2	-	-
Unspecified histology	6	-	-
Unknown	9	-	-
Lateralization of epilepsy (Rs/Ls/Bi)	26/16/2	-	-
Lateralization of electrode implantation			
Right-sided	10	-	-
Left-sided	6	-	-
Bilateral with right hemispheric predominance	16	-	-
Bilateral with left hemispheric predominance	12	-	-
SEEG monitoring duration (day)	-	3–15	7.5 (6.8, 11)
Number of electrodes implants (*n*)	-	4–12	7.7 ± 0.4
Follow-up time (month)	42	9–51	32 (13.5, 43.3)

*Abbreviations*: *ASM* Antiseizure medication, *Bi* Bilateral, *D* Daily, *Ls* Left-sided, *M* monthly, *Rs* Right-sided, *W* Weekly

The onset symptoms of seizures observed in patients included confusion, oropharyngeal automatisms, nausea, palpitations, fear, and other complex partial seizures, which may occur with or without generalized tonic–clonic seizures (GTCS). Secondary manifestations of GTCS included head-eye deviation, involuntary movements of both upper limbs, hand ball-like movements, and involuntary body rotation, often followed by loss of consciousness.

### Electrode implantation design

The electrode implantation plan was based on the patient’s seizure symptoms, 24-h video electroencephalogram (VEEG) data, neuroimaging findings (3-dimensional T1-weighted sequence [3D-T1], PET), and other non-invasive evaluation results. These data were used to hypothesize the location of the epileptic network and to define the intracranial electrode coverage area. The hypothesized preoperative epileptic foci identified for SEEG included the temporal lobe (20 cases), frontal lobe (12 cases), parietal lobe (1 case), multiple lobes (8 cases), and cingulate gyrus (3 cases). Preoperative imaging included 3D-enhanced thin-layer MRI and thin-layer CT scans (1-mm slice thickness and interval), with imaging data integrated into the neurosurgery stereotactic surgical assistance system workstation (SINOVATION, Sinovation Medical Technology Co., Ltd, Beijing, China) for multimodal image fusion. A multi-channel electrode implantation plan was then designed. During the five-year period, the department employed the Leksell stereotactic headframe (ELKTA Ltd., Stockholm, Sweden) and surgical robots (SINO ROBOTICS, Sinovation Medical Technology Co., Ltd, Beijing, China) for electrode implantation.

### Electrode implantation

The Leksell stereotactic headframe (ELKTA Ltd., Stockholm, Sweden) and SINO ROBOTICS (Sinovation Medical Technology Co., Ltd, Beijing, China) were employed for electrode implantation.The Talairach's stereotactic implantation method was utilized, involving the insertion of multi-contact electrodes from SINO ROBOTICS (Sinovation Medical Technology Co., Ltd, Beijing, China) into the brain. Standard electrodes featured either 6 or 16 contacts, with each contact measuring 2 mm in length and 0.8 mm in diameter, separated by 1.5 mm of intercontact space. The electrode positions were determined based on the hypothesized epileptogenic zones identified during the first-stage evaluation. Postoperative computed tomography (CT) and/or MRI scans were conducted to confirm the absence of complications, such as intracranial hemorrhage, and to verify the accuracy of electrode placement. The anatomical trajectory of the electrodes was reviewed using the Huake Precision Software (SINOVATION, Sinovation Medical Technology Co., Ltd, Beijing, China) for CT/MRI image registration. SEEG recordings were obtained using the Nihon Kohden system (Nihon Kohden, Inc., Tokyo, Japan), capturing at least three habitual seizures from patients over a period of 1 to 3 weeks.

### SEEG monitoring and cortical electrical stimulation (mapping)

SEEG signals were recorded using the Nihon Kohden system at a 1024 Hz sampling rate with 16-bit resolution. The system included a hardware high-pass filter (cut-off at 0.16 Hz) and an antialiasing low-pass filter (cut-off at 70 Hz). Brain regions exhibiting focal low-amplitude fast rhythms on low-frequency rhythms were identified as typical seizure onset sites. After capturing three or more habitual seizures, cortical electrical stimulation was conducted to identify functional areas. Stimulation parameters commonly used included low-frequency (0.9 Hz) and high-frequency (50 Hz) stimulation, with a pulse width of 200–300 μs, intensity of 1–12 mA, duration of 2–5 s per stimulation, and intervals of 10–20 s between stimulations. During electrical stimulation, if habitual seizures were elicited at lower current intensities (1–4 mA) and the corresponding key electrode contacts matched the seizure initiation area recorded by SEEG, these contacts were confirmed as the epileptic zone (EZ) and used as targets for RF-TC. Contacts selected for RF-TC were determined based on four criteria [8–10]: (1) sampling structures in the EZ identified through visual analysis and quantitative SEEG analysis (e.g., Epileptogenicity Index); (2) sampling structures in the early propagation zone; (3) contacts located at or near MRI-visible lesion borders suspected to be epileptogenic; and (4) induction of habitual ictal clinical phenomena during stimulation of these contacts. Thermal coagulation was avoided if the target contact was near functional areas or closely associated with blood vessels. The RF-TC device (Model No. R2000B-M1, BNS, Beijing, China) was set to deliver 7.5 W of power in two 30-s sessions with a 30-s pause between sessions. No significant discomfort was reported during the procedure, although some patients described hearing abnormal intracranial sounds. About 24 h post-procedure, some patients experienced tolerable headaches, possibly linked to brain edema near the RF-TC-treated tissue. All patients were discharged without significant new neurological deficits. The timing of surgical resection following RF-TC depended on seizure frequency: patients with daily seizures were observed were for one week, and those with weekly seizures were observed for three months. Surgical intervention was recommended if seizure frequency did not reduce by 50%.

### Follow up and evaluation methods

A monthly seizure frequency was recorded for each patient after thermocoagulation, and a reduction in seizure frequency was calculated relative to the baseline frequency prior to RF-TC. Patients who underwent resection surgery were followed up until the time of surgery, while those who did not were followed up until August 2024. The modified Engel classification system was used to evaluate treatment efficacy: Engel I represented seizure cessation or occasional non-disabling simple partial seizures; Engel II indicated a reduction in seizure frequency by more than 90%, with occasional complex partial seizures; Engel III corresponded to a 50–90% reduction in seizure frequency; and Engel IV represented a reduction in seizure frequency of less than 50%.

### Statistical methods

Data were analyzed using SPSS version 25. Self-paired quantitative data were employed to compare treatment efficacy before and after RF-TC guided by SEEG, using paired *t*-tests. Paired comparison rank sum tests were conducted, with statistical significance defined as *P* < 0.05.

## Results

A total of 44 patients were included in the study, comprising 31 males and 13 females. The mean age of the patients was 28.6 years (range: 22.0–37.5) and the average age at seizure onset was 20.6 ± 1.9 years, with the average disease duration of 6.5 years (interquartile range: 0.8–11.3). The frequency of epileptic seizures was reported as 10 episodes per day, 13 per week, and 21 per month. The median number of medications taken was 2.0 (range: 1.0–3.0). Among the patients, one had a history of febrile seizures, one had prenatal exposure to carbon monoxide poisoning, four suffered from head trauma with intracranial hemorrhage, and one had undergone prior intracranial surgery.

### Side of electrode implantation

Ten patients had implantation on the right side, 6 on the left side, 16 had bilateral implantation with right hemispheric predominance, and 12 patients had bilateral implantation with left hemispheric predominance. The duration of SEEG monitoring averaged 7.5 days (range: 6.8–11), and the number of implanted electrodes was 7.7 ± 0.4. The average follow-up period after RF-TC was 42 months (range: 25.5–53.3).

### Follow-up outcomes after RF-TC

At 3 months post-RF-TC, 11 patients achieved Engel I and 14 achieved Engel II, accounting for 56.8% of the cohort, with 11 patients seizure-free, representing a seizure-free rate of 25%. A total of 38 patients (86.4%) experienced a reduction in seizure frequency greater than 50%. Engel III and IV grades were observed in 13 and 6 cases, respectively.

At 6 months post-RF-TC, there were 9 patients each in Engel I and II, representing 40.9% of the cohort.Among these, 9 patients were seizure-free, yielding a seizure-free rate of 20.5%. A total of 32 patients (72.7%) experienced a reduction in seizure frequency exceeding 50%, with Engel III and IV grades in 14 and 7 cases, respectively. Five patients underwent surgery during this period.

At 12 months post-RF-TC, 5 patients achieved Engel I and 13 Engel II, collectively accounting for 40.9%. Seizure-free status was observed in 5 patients, with a seizure-free rate of 11.4%. A total of 27 patients (61.4%) demonstrated a reduction in seizure frequency greater than 50%. Engel III and IV grades were recorded in 9 and 5 cases, respectively. By this time, 12 patients had undergone surgery.

At 24 months post-RF-TC, 3 patients achieved Engel I and 6 achieved Engel II, comprising 20.5% of the cohort. Seizure-free status was observed in 3 patients, representing a seizure-free rate of 6.8%. A total of 15 patients (34.1%) experienced a reduction in seizure frequency exceeding 50%. Engel III and IV grades were observed in 6 and 5 cases, respectively. By this time, 16 patients had undergone surgery.

A statistically significant difference was noted in the frequency of seizures before and after thermocoagulation among the 44 patients (*P* = 0.007). However, the effectiveness of thermocoagulation showed a decreasing trend over time. Sixteen patients were followed up for an average duration of 30.0 ± 2.4 months after resection surgery. Among these, 14 patients were classified as Engel I and 2 patients as Engel II, reflecting favorable outcomes. Detailed results are available in Tables 2 and 3.

**Table 2:** Clinical data of 44 patients with RF-TC

Patients	Sex/ Age(Years old)	Number of electrodes/Side	Hypothetical EZ/Side	Number of RF-TC electrodes	Follow up time after RF-TC(Months)	Frequency of epileptic seizures before RF-TC(times per month)	Frequency of epileptic seizures after RF-TC((times per month)	Reduction rate of seizures	Engel grading after RF-TC(3/6/12/24 months)
1	M/32	4/R,1/L	MFG/R	3/R	59	2	0	100%	I/-/-/-
2	F/24	5/R	SFG, CG/R	3/R	48	1	1	0	IV/IV/IV/IV
3	M/25	8/R	MTL/R	3/R	49	30	1	96.70%	I/I/II/II
4	M/27	6/R,2/L	MTL/R	2/R	59	12	4	66.70%	II/II/II/III
5	M/27	5/R,1/L	MFG/R	3/R	48	8	2	75%	II/II/II/-
6	F/38	2/R,8/L	MTL/Bi	4/L	52	4	3	25%	IV/IV/IV/IV
7	M/14	5/R,1/L	MFG/R	2/R	49	120	30	75%	II/III/III/-
8	M/27	5/R,4/L	ITG, Hi/R	3/R	57	1	1	0	IV/IV/IV/IV
9	M/24	4/R	SFG/R	2/R	64	4	2	50%	III/III/-/-
10	F/30	7/R	MTG, ITG/R	7/R	62	4	0	100%	I/I/II/II
11	F/26	1/R,3/L	MTG, ITG/L	3/L	63	3	2	33.30%	III/II/II/-
12	F/15	9/R,2/L	ITG, Hi/R	4/R	56	90	60	33.30%	III/III/III/III
13	M/36	2/R,5/L	ITG, Hi/L	3/L	52	15	2	86.70%	II/II/II/-
14	F/33	9/R,2/L	MFG/R	2/R	57	4	2	50%	II/-/-/-
15	M/23	8/R,1/L	PL/R	3/R	44	2	1	50%	III/III/-/-
16	M/44	4/R,4/L	MFG/L	3/L	46	8	3	62.50%	II/II/II/II
17	M/29	5/L	IFG, insula, STG/L	3/L	54	3	2	33.30%	III/III/III/III
18	M/46	5/R	MFG/R	4/R	50	4	3	25%	III/III/III/III
19	F/41	4/R,1/L	MTL, insula, TP/R	4/R	55	5	2	60%	II/II/II/III
20	M/13	1/R,3/L	MTL/L	1/R,3/L	50	1	1	0	IV/IV/IV/IV
21	F/38	5/R	CG/R	4/R	47	3	2	33.30%	III/-/-/-
22	M/50	2/R,2/L	SFG/R	2/R	38	2	0	100%	I/-/-/-
23	F/48	2/R,4/L	MTL/L	3/L	40	3	1	66.70%	II/-/-/-
24	M/14	6/L	SFG, CG/L	2/L	37	2	0	100%	I/I/I/I
25	M/30	2/R,7/L	MTL/L	3/L	32	2	1	50%	II/-/-/-
26	F/30	7/R	SFG/R	4/R	39	90	1	98.90%	I/I/I/I
27	M/22	8/R	MTL/R	2/R	37	60	45	25%	III/III/-/-
28	M/32	5/R,1/L	MTL/R	3/R	42	2	2	0	IV/IV/-/-
29	F/38	7/L	MTL, TP/L	3/L	34	90	10	88.90%	I/I/II/II
30	M/22	9/R,1/L	MFG/R	2/R	34	2	1	50%	III/III/III/III
31	M/22	8/R	IFG, insula/R	2/R	36	2	3	0	IV/IV/IV/IV
32	M/39	6/R,1/L	MTL/R	3/R	27	4	0	100	I/I/II/II
33	M/27	11/L	PL and OL/L	3/L	26	120	20	83.30%	II/II/II/II
34	F/55	5/R,2/L	MTL/R	2/R	22	6	2	66.70%	II/III/III/-
35	M/17	2/R,8/L	SFG, CG/L	2/L	23	4	1	75%	II/II/II/-
36	M/18	2/R,8/L	IFS/L	3/L	22	30	1	96.70%	I/I/I/-
37	M/12	1/R,7/L	CG, OFG/L	4/L	24	300	0	100%	I/I/I/I
38	M/12	1/R,8/L	CG, SFG/L	5/L	22	30	10	66.70%	II/III/-/-
39	M/39	6/R,1/L	CG, OFG/R	4/R	20	4	2	50%	II/II/II/-
40	M/20	10/R	CG, SFG/R	3/R	15	150	2	98.70%	I/I/I/-
41	M/25	10/L	MTL, ITG, TP/L	4/L	14	2	1	50%	III/III/III/-
42	F/32	1/R,11/L	MTL/L	3/L	13	2	1	50%	III/III/III/-
43	M/31	5/R,4/L	MTL/R	3/R	13	2	1	50%	III/III/III/-
44	F/13	10/L	TL and OL/L	2/L	9	3	1	66.70%	III/III/-/-

*Abbreviations*: *M* Male, *F* Female, *R* Right, *L* Left, *EZ* Epileptic zone, *RF-TC* Radiofrequency thermocoagulation, *M* Month, *F* Frequency, *SFG* Superior frontal gyrus, *MFG* Middle frontal gyrus, *IFG* Inferior frontal gyrus, *CG* Cingulated gyrus, *OFG* Orbitofrontal gyrus, *STG* Superior Temporal Gyrus, *MTG* Middle temporal gyrus, *ITG* Inferior temporal gyrus, *MTL* Mesial temporal lobe, *Hi* Hippocampus, *TL* Temporal lobe, *PL* Parietal lobe, *OL* Occipital lobe

**Table 3:** Preoperative evaluation and resection efficacy of 16 patients with RF-TC

Patients	MRI	PET	Symptomatology	Engel grade of epilepsy after resection	Pathology
1	Right frontal lobe	Bilateral frontal and parietal lobes	GTCS	I	FCDIIb
2	Right frontal lobe	Negative	Head and eyes turning to left	I	Neuronal degeneration and necrosis
3	Right temporal lobe	Right temporal lobe	Absence seizure, eyes turning to left, Stiffness of both upper limbs	I	Gliosis
4	Negative	Negative	GTCS	I	Gliosis like Hair cell
5	Left temporal lobe	Left temporal lobe	eyes turning to left, loss of consciousness	II	Left temporal arachnoid cyst
6	Negative	Right temporal lobe	Uncontrolled movements of both upper limbs and hands➩GTCS	I	Gliosis, Hippocampal sclerosis
7	Left Hippocampus	Left Hippocampus	clonic seizure of right limb and hand➩GTCS	I	Gliosis, Hippocampal sclerosis
8	Negative	Right frontal lobe	convulsion of the limbs, Upward gaze with both eyes, Drooping corners of the mouth	I	FCDIIa
9	Negative	Negative	GTCS	I	Neuronal degeneration and necrosis
10	Right MCC	Right MCC	GTCS	I	Pilocytic astrocytoma
11	Right frontal lobe	Right frontal lobe	GTCS	I	Neuronal degeneration and necrosis
12	Left Hippocampus	Left Hippocampus	convulsion of the limbs, loss of consciousness, Involuntary movement of mouth and limbs	I	Gliosis, Hippocampal sclerosis
13	Left temporal lobe	Left temporal lobe and Hippocampus	Absence seizure, Oropharyngeal automatism, Grope with both hands➩GTCS	I	Gliosis, Hippocampal sclerosis
14	Right Hippocampus	Right temporal lobe and Hippocampus	loss of consciousness, chew	I	Neuronal degeneration and necrosis, Gliosis, Hippocampal sclerosis
15	Right Hippocampus	Right Hippocampus	Absence seizure➩GTCS	I	Hippocampal atrophy
16	Multiple intracranial nodules	Multiple intracranial nodules	Absence seizure	II	Confusion of cortical neurons

*Abbreviations*: *MRI* Magnetic resonance imaging, *PET* Positron emission computed tomography, *FCD* Focal cortical dysplasia, *GTCS* Generalizedtonic-donicseizures

## Discussion

In recent years, various minimally invasive techniques have been employed to treat drug-resistant epilepsy, including magnetic resonance imaging-guided laser ablation therapy [11], high-focused ultrasound [12], and SEEG-guided RF-TC. RF-TC, performed under SEEG guidance, does not impose additional surgical risks or costs and allows targeted thermocoagulation of adjacent contacts based on SEEG-recorded seizure initiation zones. Its safety has been corroborated by numerous studies [13, 14].

Due to the assumption that SEEG is based on the preoperative epileptic zone, the implantation plan of SEEG and the accuracy of intraoperative electrode implantation may affect the results of RF-TC. Previous literature has reported significant variability in the effectiveness of RF-TC guided by SEEG. A recent meta-analysis that included six studies [4, 5, 15–18] analyzed data from 296 patients, revealing incidence-free rates after one year ranging from 4 to 71%. Using a random-effects model, the incidence-free rate and response rate (defined as a reduction in seizures by more than 50%) after one year of surgery were calculated as 23% (95% CI: 8% to 50%) and 58% (95% CI: 36% to 77%), respectively. In a 10-year follow-up study on RF-TC [4], 25% of patients were seizure-free at two months, and 7% were seizure-free at one year. The response rates were reported as 67% at two months, 48% at one year, and 13% at 10 years post-surgery. Therefore, it can be concluded that the effectiveness of RF-TC decreases gradually over time.

In this study, it can be seen that as the follow-up time prolongs, the seizure free rate and response rate of RF-TC continue to decrease. This is basically consistent with previous research results.

At 3 months post-RF-TC, this study observed a seizure-free rate of 25% (11 patients) and a response rate of 86.4% (38 patients), which surpasses outcomes reported in previous international studies [4]. This improved outcome may be attributed to advancements in the conceptualization of epilepsy networks, stereotactic technologies, and electrode implantation accuracy. The evolution of SEEG techniques has integrated the effectiveness of RF-TC into the preoperative electrode implantation strategy, leading to dense electrode coverage over suspected EZ and their surroundings to enhance RF-TC outcomes. For instance, in this study, three patients (cases 36, 37, and 40) underwent comprehensive electrode implantation targeting the suspected EZ during the preoperative SEEG pathway design. These patients remained seizure-free for three months post-RF-TC and continued to achieve Engel I classification at the one-year follow-up. 

Additionally, the short-term effectiveness of SEEG-guided RF-TC serves as a reliable predictor of accurate EZ localization. A study by Bourdillon et al. [13] demonstrated that responders to RF-TC within two months post-treatment exhibited a 93% positive predictive value for achieving Engel I or II following subsequent lesion resection. This highlights that short-term efficacy of RF-TC can indicate the precision of SEEG in identifying EZs. Among the 16 patients in this study who underwent surgical resection, the follow-up period averaged 30.0 ± 2.4 months post-resection revealing 14 patients achieved Engel I, including 7 patients who were seizure-free three months after RF-TC. These findings underscore the utility of good short-term RF-TC outcomes in predicting the accuracy of EZ localization, which can guide future surgical planning and improve long-term seizure control.

Based on anatomical localization and direct electrical stimulation results with SEEG, potential functional impairments caused by SEEG-guided RF-TC can often be anticipated. In this study, one patient experienced a transient functional impairment post-RF-TC, which resolved completely before discharge. In a follow-up study by Bourdillon et al. [4], RF-TC was associated with a permanent functional defect rate of 1.1% and a temporary defect rate of 2.4%. Similarly, a meta-analysis of six studies reported a permanent functional impairment rate of 2.5% [13]. Notably, no cases of permanent functional impairment were observed in this study, suggesting a favorable safety profile for SEEG-guided RF-TC in the treatment of drug-resistant epilepsy.

In studies of etiology, SEEG-guided RF-TC has demonstrated satisfactory outcomes in treating periventricular nodular heterotopia (PNH), with 38% (95% CI: 6–84%) of PNH patients achieving seizure freedom and 83% (95% CI: 57–96%) showing a positive response [19, 20]. However, in patients with hippocampal sclerosis, the seizure-free rate for RF-TC is only 25%, compared to an 80% seizure-free rate achieved through anterior temporal lobe resection. Consistent with these findings, the current study also indicates that RF-TC is less effective for hippocampal sclerosis than anterior temporal lobectomy. Among the 16 patients who underwent resection surgery, pathological results predominantly revealed hippocampal sclerosis and focal cortical dysplasia (FCD). Consequently, RF-TC guided by SEEG is not recommended as a first-line treatment for hippocampal sclerosis [21]. The efficacy of RF-TC appears to be more favorable in treating gray matter nodular ectopia compared to FCD [18, 22]. For different pathological subtypes of FCD, the EZ may vary in extent. In cases with wider lesion ranges, RF-TC's limited thermocoagulation area may not adequately control seizures. For large or complex FCD cases, multi-point thermocoagulation has been employed to reduce seizures and guide subsequent treatments [23]. According to French SEEG guidelines, RF-TC is a selective treatment option for deep gray matter ectopia and hypothalamic hamartoma [22]. It is also considered as a primary treatment alternative in high-risk cases where resection may result in significant functional deficits [4]. Despite these advancements, clinical studies remain insufficient regarding the application of RF-TC for conditions with diffuse epileptic networks or unclear localized EZ, such as viral encephalitis or hypoxic-ischemic encephalopathy. For cases where EZ are relatively localized, and resection poses significant risks, SEEG-guided RF-TC remains an effective treatment option. Further research is needed to validate its efficacy in broader pathological contexts.

The limitations of this study include the small sample size, uneven distribution of epilepsy etiologies, and variability in follow-up durations. Additionally, the lack of consensus on the optimal timing for surgical resection following RF-TC complicates the assessment of RF-TC's long-term efficacy. In this study, some patients underwent EZ resection within 3–6 months after RF-TC, further limiting the evaluation of RF-TC's sustained therapeutic effects.

## Conclusions

The design of SEEG-guided RF-TC relies on an anatomy-electro-clinical framework, with its effectiveness contingent upon accurate hypotheses regarding the EZ. While electrodes densely surrounding the EZ allow for precise thermocoagulation, the limited range of thermal damage from SEEG electrodes prevents the achievement of long-term seizure control, restricting RF-TC to a palliative surgical therapy option [24]. Nonetheless, RF-TC effectively predicts the accuracy of SEEG in localizing the EZ, reduces seizure frequency, and provides significant benefits for epilepsy patients.

The findings of this study affirm that SEEG-guided RF-TC is a safe and effective therapeutic approach. However, the small sample size and significant variability in follow-up durations limit the robustness of conclusions. Future studies should aim to include larger sample sizes and extend post-RF-TC follow-up periods to better evaluate the potential early-stage benefits of this technique for epilepsy patients.

## Supplementary Information

Supplementary Material 1.

Supplementary Material 2.

Supplementary Material 3.

Supplementary Material 4.

Supplementary Material 5.

## Abbreviations

DRE: Drug resistant epilepsy
EZ: Epileptic zone
GTCS: Generalized tonic–clonic seizures
FCD: Focal cortical dysplasia
PNH: Periventricular nodular heterotopia
RF-TC: Radiofrequency thermocoagulation
SEEG: Stereotactic electroencephalography
VEEG: Video electroencephalogram
