# Supplementary material for: Whether radiofrequency thermocoagulation guided by stereotactic electroencephalography can benefit drug-resistant epilepsy in the early follow-up stage
Source: Acta Epileptol. 2025 Mar 5;7:16. doi: 10.1186/s42494-025-00207-5 (PMC11960330; doi:10.1186/s42494-025-00207-5)
Supplement: Supplementary file 1 — Supplementary Material 1. [file 42494_2025_207_MOESM1_ESM.docx]

**Whether radiofrequency thermocoagulation guided by SEEG stereotactic electroencephalography can benefit drug-resistant epilepsy in the early follow-up stage**

**Informed Consent Form**

**Dear Patients / Participants:**

You are now invited to participate in a study entitled "**Whether radiofrequency thermocoagulation guided by SEEG stereotactic electroencephalography can benefit drug-resistant epilepsy in the early follow-up stage**". Before you participate in this study, please read the informed consent form carefully and make your decision on whether to participate in this study. You can ask your doctor / investigator about anything you do not understand and let him / her explain to you until you fully understand. You can fully discuss it with your family and friends before making your decision to participate in this study. If you are participating in another study, please inform your doctor or investigator. The main contents of this study are as follows.

**1. Background:**

1.This study was led by Tianjin University and jointly organized and implemented with Tianjin Huanhu Hospital (School of Neurology and Neurorehabilitation, Tianjin Medical University). The project period was from December 2021 to November 2024.

2.The study purpose is to study the stereotactic electroencephalography on the treatment effect, so we need to review the relevant data before and after the treatment (including your age, gender, epilepsy duration, seizure type, medication, stereotactic electroencephalography monitoring data, imaging data, postoperative pathology, etc.) for research work.

3. This study has been approved by the ethics committee of our institute, which is an organization that protects the rights of the patients / participants.

**2. Design and Process:**

1.This study will collect patients treated with radiofrequency thermocoagulation guided by SEEG stereotactic electroencephalography in Tianjin Huanhu Hospital from 2018 to 2023, and retrospectively analyze the early treatment effects of patients (9 months-24 months)

2.If you volunteer to participate in this study, we will need you to do the following: we need to collect relevant information before and after your treatment (including your age, gender, epilepsy duration, seizure type, medication, stereotactic electroencephalogram monitoring data, imaging data, postoperative pathology, etc.), will not affect your normal diagnosis and treatment.

3. After we collect these data, we will organize, analyze and conduct statistical research, analyze the results of these data, and draw the corresponding results and conclusions.Finally,the results and conclusions will be published in a paper.

4. Your information is stored in the form of electronic version in a special computer, this computer is not used for other purposes, and has a password, only the personnel who carried out this study (1 person) have this password. Your medical records will be kept in the hospital for access only by researchers and, if necessary, members of the government administration or institutional review board of Tianjin Huanhu Hospital may have access to your personal data. The results will be published in statistically analyzed data and will not contain any identifiable patient / participant information.

**3. Possible risks and benefits:**

1. Possible risks: This study is retrospective and will not interfere with your diagnosis and treatment. The whole study process is subject to the supervision of the ethics committee of Tianjin Huanhu Hospital. If you encounter any questions during the research process, you can consult the study doctor or the ethics committee.

2. Possible benefits: Since this study is retrospective and the results may not be directly used for your diagnosis and treatment, you may be reimbursed for participating in this study, but you are not paid. However, the analysis of your treatment effect will help to make a clear diagnosis or effective treatment of refractory epilepsy in the future, and improve the cure rate of stereotactic EEG-guided radiofrequency thermocoagulation for refractory epilepsy. We hereby thank you for your participation in scientific research and for your contributions to the development of medicine!

**4. voluntary participation**

Your participation in the trial is completely voluntary and you can withdraw at any time without any reason, which will not affect your relationship with medical staff and future treatment.

**5. Research expenditure**

Participation in this study will not increase any cost to you. All study costs will be resolved by the investigator.

**6. Contact person and contact information**

If you have any questions about this study, you can contact the subject contact person directly at 022-59065274. Mobile phone: 13994709646. If you have any questions related to the subject, or you want to reflect the difficulties, dissatisfaction or concerns encountered during the participation in this study, or want to provide opinions and suggestions related to this study, please contact the Ethics Committee of Tianjin Huanhu Hospital, tel: 022-59065828, email: tjhh_ec@163.com.

**Informed Consent Form signature**

As a participant, I read the above information and understand the purpose of the study and the potential benefits of the study, and I have received satisfactory answers to all the questions raised by the procedures and content of the study. I agree to contribute my relevant information and provide my relevant health information for research and development work. I volunteered to sign this informed consent form and voluntarily participate in the study.

**Participant Signature: Signature Date:**

**Signature of the legal agent (if necessary) Date: (if necessary)**

**Witness Signature (if necessary) Date: (if necessary)**

We have read and explained this informed consent form to the study subject, and answered all the questions he / she asked. He / she herself has understood and agreed to participate in this scientific study.

**Investigator Signature: Signature Date:**
