# Supplementary material for: Whether radiofrequency thermocoagulation guided by stereotactic electroencephalography can benefit drug-resistant epilepsy in the early follow-up stage
Source: Acta Epileptol. 2025 Mar 5;7:16. doi: 10.1186/s42494-025-00207-5 (PMC11960330; doi:10.1186/s42494-025-00207-5)
Supplement: Supplementary file 3 — Supplementary Material 3. [file 42494_2025_207_MOESM3_ESM.pdf]

天津市环湖医院伦理委员会伦理审查批件

用钉钉扫码

天津市环湖医院　　创建时间：2024-07-22

审批编号	202407221548000253069
创建人	王丽琳
创建人部门	职能科室-科教科-伦理办公室
批件号	（津环）伦审第（2024-173）号
项目名称	人工酶电极的临床应用研究
项目来源	国家重点研发计划-天津市环湖医院
研究单位	天津市环湖医院
承担科室	神经外科
主要研究者	佟小光
职称	主任医师
审查类别	初始审查
审查方式	会议审查
审查日期	2024-07-18
审查地点	第三会议室
出席人数	11人（其中0人回避）
审查委员	王金环　闫华　刘晓民　陈蕾　张毅　张金玲　王宏图　靳松　金奕　舒向荣　李明宇
批准文件	1　研究材料诚信承诺书<br>2　伦理审查申请表<br>3　研究人员信息、研究所涉及的相关机构的合法资质证明以及研究经费来源说明<br>4　研究方案<br>5　知情同意书<br>6　信息数据的来源证明<br>7　科研立项证明<br>8　利益冲突申明<br>9　招募广告及其发布形式<br>10　研究成果的发布形式说明
审查意见	批准<br>根据《涉及人的生物医学研究伦理审查办法》、《药物临床试验伦理审查工作指导原则》、《药物临床试验质量管理规范》（GCP）、《涉及人的临床研究伦理审查委员会建设指南》、世界医学会《赫尔辛基宣言》《医疗器械临床试验规定》、《体外诊断试剂临床研究技术指导原则》、和CIOMS《人体生物医学研究国际道德指南》的伦理原则，经本伦理委员会审查，同意按所批准的文件开展本项研究。<br>请遵循GCP原则、遵循伦理委员会批准的方案开展临床研究，保护受试者的健康与权利。<br>研究过程中若变更主要研究者，对临床研究方案、知情同意书、招募材料等的任何修改，请申请人提交修正案审查申请。<br>请按照伦理委员会规定的年度/定期跟踪审查频率，按期提交研究进展报告；申办者应当向组长单位伦理委员会提交各中心研究进展的汇总报告；当出现任何可能显著影响试验进行、或增加受试者危险的情况，以及所有可疑且非预期严重不良反应；可能对受试者的安全或者临床研究的实施产生不利影响的新信息时，请及时报告伦理委员会请申请人及时向伦理委员会提交书面报告。<br>研究纳入了不符合纳入标准或符合排除标准的受试者，符合中止试验规定而未让受试者退出研究，给予错误治疗或剂量，给予方案禁止的合并用药等没有遵从方案开展研究的情况；发生为消除对受试者紧急危害的研究方案的偏离或者修改，或可能对受试者的权益/健康、以及研究的科学性造成不良影响等违背GCP原则的情况，请申办者/

	研究者提交违背方案报告。<br>申请人暂停或提前终止临床研究，请及时提交暂停/终止研究报告。<br>完成临床研究，请申请人提交研究完成报告。
年度/定期跟踪审查频率	12个月
有效期	1年
说明	自批准之日起，如有效期内试验逾期未实施需提出延长有效期申请。
联系人	陈亚星
联系电话	022-59065828
主任委员/副主任委员	王金环
伦理委员会	天津市环湖医院医学伦理委员会（盖章）
日期	2024-07-22
审批流程	同意<br>王金环 已同意 签名：王金环 2024-07-30 20:05:15
	抄送 王丽琳,陈亚星 2024-07-30 20:05:15

打印时间：2024-07-31 10:39 打印人：王丽琳
