# Supplementary material for: Whether radiofrequency thermocoagulation guided by stereotactic electroencephalography can benefit drug-resistant epilepsy in the early follow-up stage
Source: Acta Epileptol. 2025 Mar 5;7:16. doi: 10.1186/s42494-025-00207-5 (PMC11960330; doi:10.1186/s42494-025-00207-5)
Supplement: Supplementary file 4 — Supplementary Material 4. [file 42494_2025_207_MOESM4_ESM.docx]

**立体定向脑电图引导下的射频热凝是否能改善药物难治性癫痫的早期效果**

**知情同意书（INFORMED CONSENT FORM）**

**尊敬的患者/参加者：**

现邀请您参加一项研究，题目是“**立体定向脑电图引导下的射频热凝是否能改善药物难治性癫痫的早期效果**”。在您参加此项研究之前，请您仔细阅读这份知情同意书并慎重做出是否参加本项研究的决定。您可以向您的研究医生/研究人员询问任何您不懂的地方，让他/她给您解释，直到您完全理解为止。您在做出参与此项研究的决定之前，可以和您的家人及朋友进行充分的讨论。若您正在参加别的研究，请告知您的研究医生或者研究人员。本研究的主要内容如下。

**一、研究背景：**

1、课题的性质：本研究由天津大学牵头，和天津市环湖医院(天津医科大学神经内外科及神经康复学院)共同组织实施，课题起止时间是2021年12月 至 2024年11月

2、研究目的是为了研究立体定向脑电图引导下的射频热凝对药物难治性癫痫的治疗效果，为此我们需要回顾您进行该治疗前后的相关资料（包括您的年龄、性别、癫痫持续时间、癫痫发作类型、服用药物、立体定向脑电图的监测资料、影像学资料、术后病理等）用于研究工作。

3、此项研究已经得到我院所伦理委员会的审批，这是一个保护受试患者/参加者权益的组织。

**二、研究设计和研究过程：**

1、这项研究将搜集2018年到2023年在环湖医院进行立体定向脑电图引导下射频热凝的患者，回顾性分析患者的早期治疗效果（9个月-24个月）

2、如果您自愿参加此研究，我们将需要您做以下事宜：我们需要搜集您治疗前后的相关资料（包括您的年龄、性别、癫痫持续时间、癫痫发作类型、服用药物、立体定向脑电图的监测资料、影像学资料、术后病理等），不会影响您正常的诊断和治疗。

3、当我们收集好这些资料后，进行整理、分析、统计学研究，分析这些数据的结果，得出相应的结果和结论。最后将得到的结果进行论文发表。

4、个人隐私的保密措施：您的资料以电子版的形式保存在专门的电脑中，此电脑不做他用，并设有密码，仅进行此项研究的人员（1人）有此密码。您的医疗记录将保存在医院，仅供研究人员查阅；必要时，政府管理部门或伦理审查委员会的成员按规定可以查阅您的个人资料。研究结果将以经统计分析后的数据形式发表，不包含任何可识别的患者/参加者信息。

**三、可能的风险和受益：**

1、可能的风险：本项研究为回顾性研究，不会干扰您的诊断和治疗。整个研究过程接受天津市环湖医院伦理委员会的监督，研究过程中如遇到任何疑问可向研究医生或伦理委员会咨询。

2、可能的受益：由于此项研究为回顾性研究，其结果不一定能够直接用于您的诊断和治疗，您参加此项研究可能会得到一些费用方面的报销，但没有报酬。但是通过对您的治疗效果进行分析将有助于未来对难治性癫痫做出明确的诊断或进行有效的治疗，并提高立体定向脑电图引导的射频热凝对难治性癫痫的治愈率。在此我们为您能够参与到科学的研究，并为医学的发展所做出的贡献表示感谢！

**四、自愿参加**

您参与试验是完全自愿的，您可以随时退出而无需理由，绝不会影响您和医务人员的关系及今后的诊治。

**五、研究费用**

参加此项研究不会增加您的任何费用。所有研究费用均由研究者自行解决。

**六、联系人及联系方式**

如果您本人对此项研究有任何疑问，可直接与课题联系人联系，联系电话：022-59065274。手机：13994709646。如果您有与课题相关的任何问题，或者您想反映参与本研究过程中遭遇的困难、不满和忧虑，或者想提供与本研究有关的意见和建议，请联系天津市环湖医院伦理委员会，联系电话：022-59065828，电子邮件：tjhh_ec@163.com。

**知情同意书签字处**

作为参与者，我以阅读上述信息，并理解该研究的目的以及参见该研究带来的潜在益处，我对研究程序、研究内容提出的所有问题均已得到令我满意的答复。我同意贡献我的相关资料并提供我的有关健康信息用于研究与开发工作。我自愿签这份知情同意书，并自愿参加这项研究。

参加者签字： 签字日期：

法定代理人签字（必要时） 签字日期：（必要时）

见证人签字（必要时） 签字日期：（必要时）

我们已经向研究对象宜读和解释了这份知情同意书，并且回答了他/她所提出的所有问题。他/她本人也已经理解并同意参加此项科学研究。

研究者签字： 签字日期：
