# Supplementary material for: Whether radiofrequency thermocoagulation guided by stereotactic electroencephalography can benefit drug-resistant epilepsy in the early follow-up stage
Source: Acta Epileptol. 2025 Mar 5;7:16. doi: 10.1186/s42494-025-00207-5 (PMC11960330; doi:10.1186/s42494-025-00207-5)
Supplement: Supplementary file 5 — Supplementary Material 5. [file 42494_2025_207_MOESM5_ESM.pdf]

课题编号：2021YFF1200705　　　　　　　　　**密　　级：公开**

国家重点研发计划
课题任务书

课题名称：　人工酶电极的临床应用研究

所属项目：　新一代高相容性生物植入电极设计与应用

所属专项：　生物与信息融合（BT 与 IT 融合）

项目牵头承担单位：　天津大学

课题承担单位：　天津市环湖医院

课题负责人：　佟小光

执行期限：　2021 年 12 月 至 2024 年 11 月

中华人民共和国科学技术部制

2021 年 12 月 13 日

0003YF 2021YFF1200705 2021-12-13 10:17:24

填 写 说 明

一、任务书甲方即项目牵头承担单位，乙方即课题承担单位。

二、任务书通过“国家科技计划管理信息系统公共服务平台”，按照系统提示在线填写。

三、任务书中的单位名称，请按规范全称填写，并与单位公章一致。

四、任务书要求提供乙方与所有参加单位的合作协议，需对原件进行扫描后在线提交。

五、任务书中文字须用宋体小四号字填写。

六、凡不填写内容的栏目，请用“无”表示。

七、乙方完成任务书的在线填写，提交甲方审核确认后，用A4纸在线打印、装订、签章。一式八份报项目牵头承担单位签章，其中课题承担单位一份，课题负责人一份，作为项目任务书附件六份。

八、如项目下仅设一个课题，课题任务书只需填报课题预算部分。

九、涉密课题请在“国家科技计划管理信息系统公共服务平台”下载任务书的电子版模板，按保密要求离线填写、报送。

十、《项目申报书》和《项目任务书》是本任务书填报的重要依据，任务书填报不得降低考核指标，不得自行对主要研究内容作大的调整。《项目申报书》、《项目任务书》和本任务书将共同作为课题过程管理、综合绩效评价（验收）和监督评估的重要依据。

课题基本信息表

<table>
<tr><td>课题名称</td><td colspan="4">人工酶电极的临床应用研究</td></tr>
<tr><td>课题编号</td><td colspan="4">2021YFF1200705</td></tr>
<tr><td>所属项目</td><td colspan="4">新一代高相容性生物植入电极设计与应用</td></tr>
<tr><td>所属专项</td><td colspan="4">生物与信息融合（BT 与 IT 融合）</td></tr>
<tr><td>密级</td><td>■公开 □秘密 □机密</td><td>单位总数</td><td colspan="2">4</td></tr>
<tr><td>课题类型</td><td colspan="4">□基础前沿■重大共性关键技术□应用示范研究□其他</td></tr>
<tr><td>课题活动类型</td><td colspan="4">□基础前沿□应用研究■试验发展</td></tr>
<tr><td>课题研究所属学科</td><td colspan="4">自然科学相关工程与技术
生物医学工程学</td></tr>
<tr><td>课题成果应用的主要国民经济行业</td><td colspan="4">科学研究和技术服务业
研究和试验发展
医学研究和试验发展</td></tr>
<tr><td>课题的社会经济目标</td><td colspan="4">卫生事业发展
诊断与治疗</td></tr>
<tr><td>经费预算</td><td colspan="4">总需求 546.92 万元，其中中央财政专项资金需求 546.92 万元</td></tr>
<tr><td rowspan="2">课题周期节点</td><td>起始时间</td><td>2021 年 12 月</td><td>结束时间</td><td>2024 年 11 月</td></tr>
<tr><td>实施周期</td><td>共 36 个月</td><td>预计中期时间点</td><td>2023 年 05 月</td></tr>
<tr><td rowspan="7">课题承担单位</td><td>单位名称</td><td>天津市环湖医院</td><td>单位法定代表人姓名</td><td>刘钢</td></tr>
<tr><td>单位性质</td><td>其他事业单位</td><td>组织机构代码</td><td>40135431X</td></tr>
<tr><td>单位主管部门</td><td>天津市卫生健康委员会</td><td>隶属关系</td><td>地方</td></tr>
<tr><td>单位所属地区</td><td>天津市</td><td>地市（市、自治州、盟）</td><td>天津市 津南区</td></tr>
<tr><td>通信地址</td><td>天津市津南区吉兆路 6 号</td><td>邮政编码</td><td>300350</td></tr>
<tr><td>单位开户名称</td><td colspan="3">天津市环湖医院</td></tr>
<tr><td>开户银行（全称）</td><td>中国银行股份有限公司天津河西支行</td><td>汇入地点</td><td>天津市 天津市</td></tr>
</table>

<table>
<tr><td></td><td>银行账号</td><td colspan="3">272660064924</td><td colspan="2">银行机构代码</td><td>104110040000</td></tr>
<tr><td rowspan="6">课题负责人</td><td>姓　名</td><td>佟小光</td><td>性　别</td><td>■男　□女</td><td>出生日期</td><td colspan="2">1968-12-26</td></tr>
<tr><td>证件类型</td><td>身份证</td><td>证件号码</td><td colspan="4">130102196812261535</td></tr>
<tr><td>所在单位</td><td colspan="6">天津市环湖医院</td></tr>
<tr><td>最高学位</td><td colspan="6">■博士□硕士□学士□其他</td></tr>
<tr><td>职　称</td><td colspan="4">■正高级□副高级□中级□初级□其他</td><td>职　务</td><td>副院长</td></tr>
<tr><td>电子邮箱</td><td colspan="3">tongxg@yahoo.com</td><td>移动电话</td><td colspan="2">13820088121</td></tr>
<tr><td rowspan="3">课题联系人</td><td>姓　名</td><td>尹绍雅</td><td>电子邮箱</td><td colspan="4">yinsy@163.com</td></tr>
<tr><td>固定电话</td><td>022-59065277</td><td>移动电话</td><td colspan="4">18622139285</td></tr>
<tr><td>证件类型</td><td>身份证</td><td>证件号码</td><td colspan="4">130402196309200336</td></tr>
<tr><td rowspan="3">课题财务负责人</td><td>姓　名</td><td>贾易晔</td><td>电子邮箱</td><td colspan="4">shhyycwwjk@tj.gov.cn</td></tr>
<tr><td>固定电话</td><td>022-59065652</td><td>移动电话</td><td colspan="4">17612258290</td></tr>
<tr><td>证件类型</td><td>身份证</td><td>证件号码</td><td colspan="4">120104197310167325</td></tr>
<tr><td rowspan="4">其他参与单位</td><td>序号</td><td colspan="2">单位名称</td><td colspan="2">单位性质</td><td colspan="2">组织机构代码</td></tr>
<tr><td>1</td><td colspan="2">复旦大学附属华山医院</td><td colspan="2">其他事业单位</td><td colspan="2">12100000425006539N</td></tr>
<tr><td>2</td><td colspan="2">天津大学</td><td colspan="2">大专院校</td><td colspan="2">121000004013593210</td></tr>
<tr><td>3</td><td colspan="2">厦门隆创思科技有限公司</td><td colspan="2">私营企业</td><td colspan="2">91350200MA33ALWX0Y</td></tr>
<tr><td rowspan="2">课题参加人数</td><td rowspan="2" colspan="2">22 人。其中：</td><td colspan="5">高级职称 5 人，中级职称 1 人，初级职称 1 人，其他 15 人；</td></tr>
<tr><td colspan="5">博士学位 6 人，硕士学位 7 人，学士学位 9 人，其他 0 人。</td></tr>
<tr><td>课题简介（限500字以内）</td><td colspan="7">我国有癫痫患者近千万，其中约30%为药物治疗耐受患者，亟需神经植入电极开展诊疗活动；且植入电极对脊髓损伤、神经痛等各神经系统疾病诊疗均具有重要临床价值；而目前植入电极相容性差、灵敏度低，难以实现长期临床应用，本项目即是针对这一问题构建高相容性人工酶电极，解决行业难题、为国家重大需求服务。其中本课题研究人工酶电极的临床效能，推动人工酶电极的临床转化。研究人工酶电极对临床患者的脑电信号检测；对癫痫信号进行精准定位和高分辨的信号采集，开展致痫灶定位研究（>120 例），实现致痫灶的精准导航。提取癫痫发作特征信号，实现人工智能对特征信号分析、识别，建立癫痫发作监测、预测</td></tr>
</table>

	模型。建立深部神经刺激治疗方案，开展人工酶电极闭环反应性神经刺激治疗研究。通过人工酶电极信号与外部处理器的高速交互，开展人工酶电极对临床患者的长期病理检测，优化临床用药方案，研究脑区神经功能。通过临床生化检验和影像学检查，研究人工酶电极的生物学效应。建立人工酶电极的临床评价体系和临床应用范式

一、目标及考核指标、考核方式/方法

请填写下表。

课题目标、预期成果与考核指标表

课题目标[1]	预期成果				考核指标[2]				考核方式（方法）及评价手段[4]
	预期成果名称			预期成果类型	指标名称	立项时已有指标值/状态	中期指标值/状态[3]	完成时指标值/状态	
（限500字以内。）	**主要成果**	1	人工酶电极的临床应用研究	☐新理论 ☐新原理 ☐新产品 ☐新技术 ☐新方法 ☐关键部件 ☐数据库 ☐软件 ☑应用解决方案 ☐实验装置/系统 ☐临床指南/规范 ☐工程工艺 ☐标准 ☑论文 ☑发明专利 ☐其他________	指标 1.1 受试者数目	无	完成受试者招募、签署知情同意书	120 例以上	研究报告
					指标 1.2 人工酶电极长期应用	无	无	信号采集时间跨度 1 年以上	发表高水平论文，信号采集原始数据电子档案
					指标 1.3 癫痫发作检测预测模型	无	无	开发癫痫发作监测预测模型 2 种以上，预测准确性大于 99%，在大于 50 例的临床数据集上进行测试	发表高水平论文，第三方测试报告
					指标 1.4 人工酶电极临床应用模式	无	完成深部神经刺激试验方案	开展致痫灶定位、深部神经刺激的临床试验及患者长期管理	发表高水平论文，第三方临床应用报告

					指标 1.5 高水平论文与专利	无	无	发表高水平论文 6 篇以上，申请专利 2 项以上	发表论文申请专利
					……				
		…							
	其他成果			同上	指标				
					……				

科技报告考核指标	序号	报告类型[5]	数量	提交时间	公开类别及时限[6]
	1	年度执行情况和技术进展情况报告	1	2022 年 12 月	公开
	2	中期执行情况报告	1	2023 年 5 月	公开
	3	年度执行情况和技术进展情况报告	1	2023 年 12 月	公开
	4	课题综合绩效自评价报告	1	2024 年 11 月	公开
	5	课题最终科技报告	1	2024 年 11 月	公开
其他目标与考核指标					

备注：

1. **“课题目标”**，应从以下方面明确描述：（1）研发主要针对什么问题和需求；（2）将要解决哪些科学问题、突破哪些核心/共性/关键技术；（3）预期成果；（4）成果将以何种方式应用在哪些领域/行业/重大工程等，并拟在科技、经济、社会、环境或国防安全等方面发挥何种的作用和影响。（5）所列主要成果原则上不超过 5 项，如有其他重要成果放在“其他”成果中表述。
2. **“考核指标”**，指相应成果的数量指标、技术指标、质量指标、应用指标和产业化指标等，其中，数量指标可以为专利、产品等的数量，论文代表作应注重质量，不以数量作为评价标准；技术指标可以为关键技术、产品的性能参数等；质量指标可以为产品的耐震动、高低温、无故障运行时间等；应用指标可以为成果应用的对象、范围和效果等；产业化指标可以为成果产业化的数量、经济效益等。同时，对各项考核指标需填写立项时已有的指标值/状态以及课题完成时要到达的指标值/状态。同时，考核指标也应包括支撑和服务其他重大科研、经济、社会发展、生态环境、科学普及需求等方面的直接和间接效益。如对国家重大工程、社会民生发展等提供了关键技术支撑，成果转让并带动了环境改善、实现了销售收入等。若某项成果属于开创性的成果，立项时已有指标值/状态可填写“无”，若某项成果在立项时已有指标值/状态难以界定，则可填写“/”。
3. **“中期指标”**，各专项根据管理特点，确定是否填写，鼓励阶段目标明确的项目课题填写中期指标。
4. **“考核方式方法”**，应提出符合相关研究成果与指标的具体考核技术方法、测算方法等。
5. **“科技报告类型”**，包括项目综合绩效评价（验收）前撰写的全面描述研究过程和技术内容的最终科技报告、项目年度或中期检查时撰写的描述本年度研究过程和进展的年度技术进展报告以及在项目实施过程中撰写的包含科研活动细节及基础数据的专题科技报告（如实验报告、试验报告、调研报告、技术考察报告、设计报告、测试报告等）。其中，每个项目在综合绩效评价（验收）前应撰写一份最终科技报告；研究期限超过 2 年（含 2 年）的项目，应根据管理要求，每年撰写一份年度技术进展报告；每个项目可根据研究内容、期限和经费强度，撰写数量不等的专题科技报告。科技报告应按国家标准规定的格式撰写。
6. **“公开类别及时限”**，公开项目科技报告分为公开或延期公开，内容需要发表论文、申请专利、出版专著或涉及技术诀窍的，可标注为“延期公开”。需要发表论文的，延期公开时限原则上在 2 年（含 2 年）以内；需要申请专利、出版专著的，延期公开时限原则上在 3 年（含 3 年）以内；涉及技术诀窍的，延期公开时限原则上在 5 年（含 5 年）以内。涉密项目科技报告按照有关规定管理。

二、课题研究内容、研究方法及技术路线

（一）课题的主要研究内容

拟解决的关键科学问题、关键技术问题，针对这些问题拟开展的主要研究内容，限 1000 字以内。

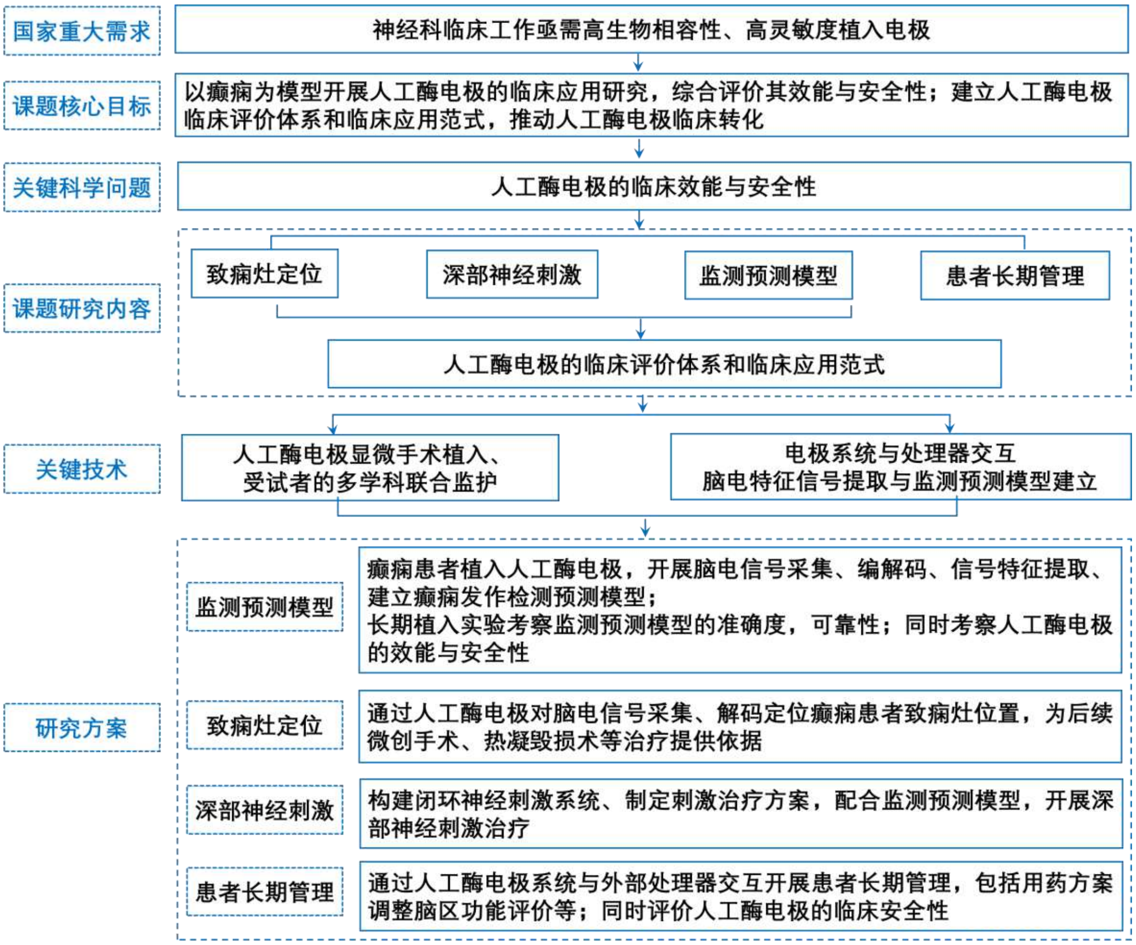

图 1. 课题五概述

课题五拟解决的关键科学问题及关键技术问题：

1) 人工酶电极临床应用的长期安全性、灵敏度、稳定性，解决目前植入电极生物相容性差、灵敏度低的痛点和难点问题。

2）建立人工酶电极的临床评价体系和临床应用范式。

3）机器学习实现对癫痫特征信号的分析、识别和预测阈值判断，建立癫痫发作检测预测模型。

课题五主要研究内容：

(1) 人工酶电极临床信号采集与癫痫监测预测模型

开展人工酶电极脑电信号采集，构建癫痫检测预测模型；评价人工酶电极的灵敏度、

精准度。招募局灶性癫痫患者（>120 例），签署知情同意书；将人工酶电极通过微创手术植入受试者大脑，开展脑电信号采集、解码；开展癫痫发作特征信号提取，机器学习实现对癫痫特征信号的分析、识别，构建特征信号库与发作预测阈值，建立癫痫发作检测预测模型；与传统监测手段进行对比研究，结合临床指标考察监测预测模型的准确性与可靠性。

(2)人工酶电极对致痫灶定位研究

开展人工酶电极致痫灶定位研究，进一步评价人工酶电极的临床精准度。通过人工酶电极对脑电信号采集、解码，对癫痫脑电信号进行定位研究，精准定位癫痫患者致痫灶位置，并与脑核磁等传统方法对比研究，考察人工酶电极对致痫灶定位的准确性、可靠性。

(3)人工酶电极深部神经刺激研究

开展人工酶电极的深部神经刺激治疗研究，考察人工酶电极临床治疗的准确性。构建闭环自反应神经刺激系统、针对病人病情制定深部刺激治疗方案，开展深部神经刺激治疗并与传统治疗及现有的临床治疗方案进行对比研究，考察治疗中受试者癫痫发作频次与强度，考察人工酶电极临床治疗中电流强度和频率调制的精准度；阐明人工酶电极对癫痫脑电信号精准调控过程；考察人工酶电极临床治疗效能。

(4)人工酶电极对患者长期管理研究及其临床安全性评价

开展人工酶电极对癫痫患者的长期管理研究，同时评价其临床安全性。建立人工酶电极系统与外部处理器的高速互联。研究人工酶电极系统与外部处理器交互开展患者长期检测，优化临床用药方案，进行脑区神经功能评价等。同时，研究临床观察与随访监护受试者健康状态，通过脑核磁、脑电图等监测受试者大脑功能状态与恢复过程。研究病人生化检查、血液学等指标；研究电极取出后的病理变化，研究人工酶电极的临床安全性。通过电子显微镜研究分子水平的病变，研究人工酶催化层在人体内的稳定性，评价人工酶电极的临床可靠性。综合评价人工酶电极的临床效能与安全性，建立人工酶电极临床评价体系和临床应用范式。

（二）课题采取的研究方法

针对课题研究拟解决的问题，拟采用的方法、原理、机理、算法、模型等

限 1000 字以内。

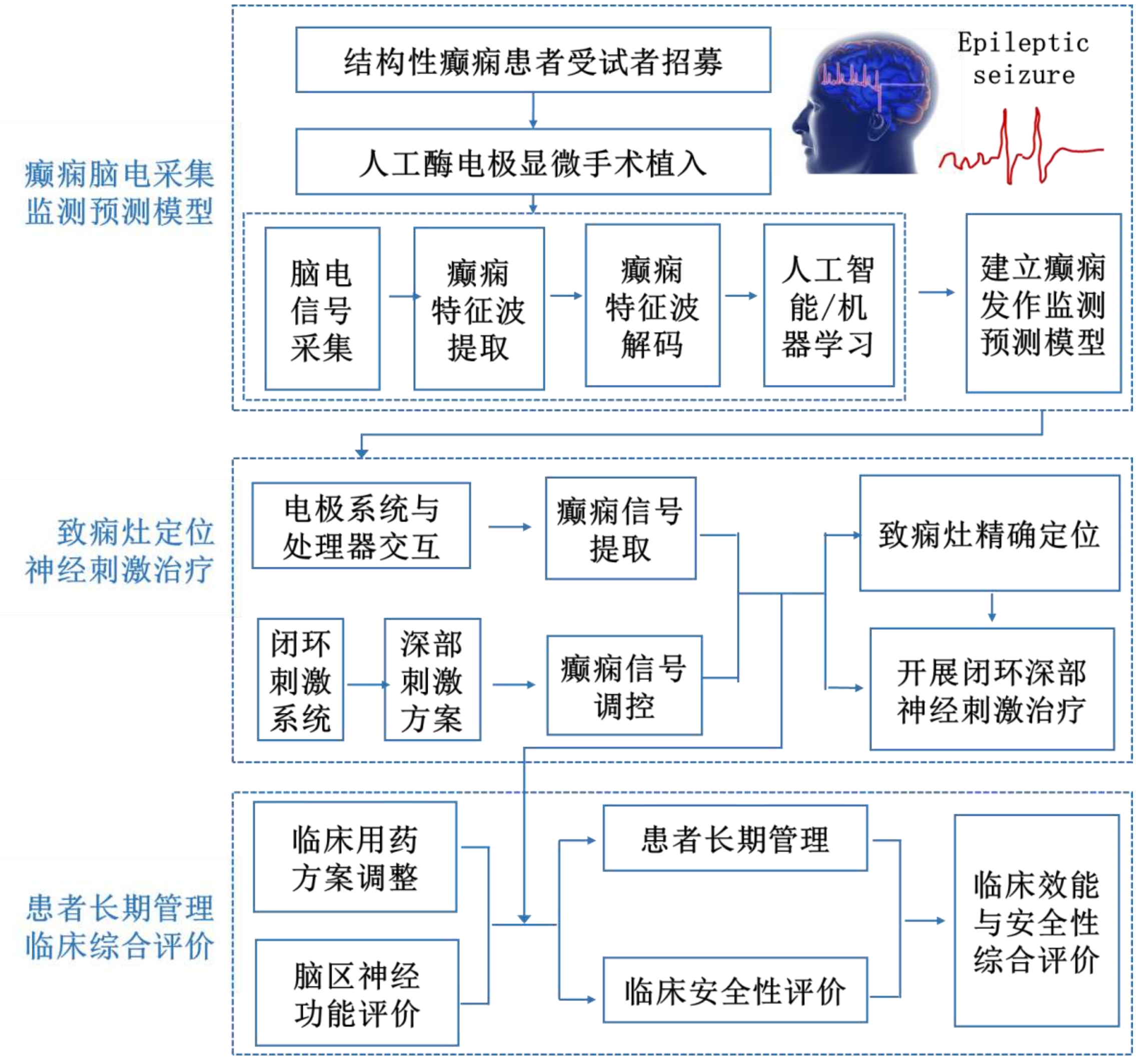

针对人工酶电极的临床应用研究，采用的研究思路方法：招募局灶性癫痫患者，对受试者植入人工酶电极，进行脑电信号采集，分析癫痫信号，对信号进行定位分析，开展致痫灶定位研究；监测受试者脑电活动情况，人工智能算法对癫痫特征信号进行分析，实现人工智能对癫痫发作信号的识别、预判和阈值判断，建立癫痫病发作监测和预测模型；开发临床用闭环神经刺激系统，制定神经刺激治疗方案，对受试者开展深部神经刺激治疗。通过长期脑电信号监测和分析，结合临床症状，开展患者进行长期管理包括对其治疗方案、用药方案经行调整，开展脑区功能评价等；同时通过临床生化检查及病理学研究考察人工酶电极的临床安全性；建立人工酶电极临床评价体系和临床应用范式，推动人工酶电极临床转化。

三、主要创新点

围绕基础前沿、共性关键技术或应用示范等层面，简述课题的主要创新点。具体内容应包括该项创新的基本形态及其前沿性、时效性等，并说明是否具备方法、理论和知识产权特征。每项创新点的描述限500字以内。

1.建立人工酶电极的临床评价体系和临床应用范式，为人工酶电极的临床转化提供理论支持奠定技术基础。

2.机器学习实现对癫痫特征信号的分析、识别和预测阈值判断，建立癫痫发作检测预测模型。为癫痫发作的预测和监测提供安全高效的新方法，为癫痫患者临床诊治服务。

3.综合评价人工酶电极临床应用的长期安全性、灵敏度、稳定性，推动人工酶电极的临床转化，确立我国在本领域的技术优势，解决目前植入电极生物相容性差、灵敏度低的痛点和难点问题。

四、预期经济社会效益

课题的科学、技术、产业预期指标及科学价值、社会、经济、生态效益。限500字以内。

科学指标：1.建立人工酶电极的临床评价体系和临床应用范式为人工酶电极的临床转化提供理论支持奠定技术基础。2.机器学习实现对癫痫特征信号的分析、识别和预测阈值判断，建立癫痫发作检测预测模型。为癫痫发作的预测和监测提供安全高效的新方法，为癫痫患者临床诊治服务。

技术指标：

表1.课题五拟完成的技术指标及其评测方法

拟完成的技术指标	评测手段/方法
受试者数目	120例以上
人工酶电极长期应用	信号采集时间跨度1年以上
癫痫发作检测预测模型	开发癫痫发作监测预测模型2种以上，预测准确性大于99%
人工酶电极临床应用模式	开展致痫灶定位、深部神经刺激的临床试验及患者长期管理
高水平论文与专利	发表高水平论文6篇以上，申请专利2项以上

科学价值：1.建立人工酶电极的临床评价体系和临床应用范式为人工酶电极的临床转化提供理论支持奠定技术基础。2.综合评价人工酶电极临床应用的长期安全性、灵敏度、稳定性，推动人工酶电极的临床转化，解决目前植入电极生物相容性差、灵敏度低的痛点和难点问题。

社会、经济、生态效益：开展人工酶生物植入电极在癫痫中的临床应用，建立癫痫发病预测、监测模型2种以上，为临床诊断和治疗效果检测提供新的技术方案。本课题的开展有望推进植入电极临床转化和产品开发，实现高相容性植入电极产业化，为临床患者服务，为国家培养相关领域人才、创造高技术工作岗位。

五、课题年度计划

按每 6 个月制定形成课题的计划进度，应将课题的考核指标分解落实到年度计划中。

按每 6 个月制定形成课题的计划进度，应将课题的考核指标分解落实到年度计划中。

1.年度：2021 年 12 月—2022 年 05 月

任务：制定临床研究方案，招募患者。制备临床样品。

考核指标：完成临床方案的制定，并提交伦理委员会。完成临床试验用人工酶电极制备。

成果形式：临床方案，人工酶电极样品。

2.年度：2022 年 06 月—2022 年 11 月

任务：开展微创手术进行人工酶电极植入，开展患者监护与随访；开展临床信号检测、致痫灶定位。

考核指标：完成患者电极植入，开展信号采集工作。

成果形式：发表论文 2 篇；预计申请专利 1 项，提供专利初步审查合格通知书。

3.年度：2022 年 12 月—2023 年 05 月

任务：通过人工酶电极对脑电信号采集,编码定位癫痫患者致病痫灶位置。

考核指标：开展致痫灶定位研究，开发癫痫发作检测预测模型。

成果形式：发表论文 2 篇；预计申请专利 1 项，提供专利初步审查合格通知书。

4.年度：2023 年 06 月—2023 年 11 月

任务：开展脑电信号采集、编解码，开展机器学习对癫痫信号特征的提取、分析、识别。继续开展癫痫发作监测预测模型研究，构建闭环神经刺激系统，制定刺激治疗方案，配合检测预测模型，开展深部神经刺激治疗。

考核指标：完成癫痫发作监测预测模型 2 种以上，预测准确性大于 99%。完成闭环神经刺激系统的设计和临床方案制定。信号采集时间跨度 1 年以上。

成果形式：发表论文 2 篇；预计申请专利 1 项，提供专利初步审查合格通知书。

5.年度：2023 年 12 月—2024 年 05 月

任务：继续进行神经刺激研究，开展患者长期管理研究，包括用药方案调整脑区功能评价等；综合评价人工酶电极的临床效能和安全性。

考核指标：完成闭环刺激系统构建和临床研究，开展患者长期管理研究。

成果形式：发表论文 2 篇；预计申请专利 1 项，提供专利初步审查合格通知书。

6.年度：2024 年 06 月—2024 年 11 月

任务：人工酶电极系统与外部处理器交互开展患者长期管理，包括用药方案调整脑区功能评价等；同时评价人工酶电极的临床安全性。总结实验，撰写结题报告。

考核指标：受试者数目 120 例以上。

成果形式：发表论文 2 篇；预计申请专利 1 项，提供专利初步审查合格通知书。

六、课题组织实施机制及保障措施

1、课题的内部组织管理方式、协调机制等，限 500 字以内。

项目实施采取首席负责制，由项目负责人佟小光教授作为主要责任人，主持项目平台的日常工作、定期召开项目会议等。设立学术委员会、管理办公室、科研执行组和监督组。在项目实施过程中实行专业分工、各负其责的管理模式。学术委员会由本领域知名专家学者组成，对项目进行咨询和技术指导；项目管理办公室协助项目负责人对项目实施进行日常事务管理，如制度颁发、会议组织、成果管理等工作；科研执行组是项目最主要的核心组成部分，负责整个项目的研究实施，控制研究进度、确保研究质量；监督组由项目内专家组成，对项目执行和经费使用全程跟踪、监督、检查等。课题承担单位天津市环湖医院充分发挥组织、协调作用，按照科技部的各项管理规定，制定各内部管理制度并监督实施；对项目中重大事项的决策进行咨询讨论；把握项目研究的国内外热点、趋势和需求；促进试验条件、数据与科研成果的内部共享，确保项目按计划顺利实施；对项目实施中出现的问题，进行协调，及时提出解决方案，并督促相关单位纠正和改进。

2、课题实施的相关政策，已有的组织、技术基础，支撑保障条件，限 500 字以内。

由课题负责人领导，项目组全体成员具体实施，并采用目标管理、定期交流、共同参与、相互协作、节点控制、平台共用等高效、有序的管理机制，开展和组织实施项目研究工作。研究团队依托医学神经生物学国家重点实验室，教育部智能医学工程中心等多个国家级、省部级平台，整合了环湖医院、华山医院、天津大学及厦门隆创思等相关领域优势单位，为项目的实施提供了丰富的仪器设备、熟练的技术人员、具备完善的资源支撑条件保障。项目团队成员学术背景覆盖多种学科，发表多篇高水平论文，在本领域具有多年工作经验是一支集成了我国在人工酶电极临床应用领域的优秀人才的研究队伍，具有项目研究目标和指标的技术保障。课题承担单位天津市环湖医院是全国著名的神经科专科医院，其神经外科是国家重点专科。具有先进的癫痫评估系统和神经影像系统、神经导航系统等；具有高水平显微外科解剖研究室；完成癫痫外科手术数千例；国内率先开展大脑半球后象限切开术、额叶切开术和颞叶切开术。国内率先开展了立体定向脑电颅内电极植入术和长程视频脑电监测、以及立体定向脑电图 SEEG 引导的致痫灶射频热凝毁损术微创治疗癫痫，为课题顺利开展奠定了良好的软硬件基础。

3、对实现项目总目标的支撑作用，及与项目内其他课题的协同机制，限 500 字以内。

本课题研究人工酶电极的临床效能，推动人工酶电极的临床转化。研究人工酶电极对临床患者的脑电信号检测；对癫痫信号进行精准定位和高分辨的信号采集，开展致痫灶定位研究，建立癫痫发作监测、预测模型；开展人工酶电极闭环反应性神经刺激治疗研究和患者长期管理研究，建立人工酶电极的临床评价体系和临床应用范式。因此本课题是对人工酶电极临床效能和安全性的综合性评价对推动人工酶电极临床转化实现项目总体目标奠定研究基础起到至关重要的作用。本课题与项目内其它课题协同开展，互相反馈，及时贡献试验结果，指导优化人工酶电极设计，共同推动项目高水平完成。课题由负责人领导并在项目学术委员会的指导下开展工作，课题负责人负责各课题间的联络与协调；课题将制定各内部管理制度并监督实施；对课题实施中重大事项的决策进行咨询讨论；把握项目研究的国内外热点、趋势和需求；促进试验条件、数据与科研成果的内部共享，确保项目按计划顺利实施；对项目实施中出现的问题，进行协调，及时提出解决方案推动项目高水平实施。

七、知识产权对策、成果管理及合作权益分配

限 500 字以内。

1. 知识产权对策

本项目按照《中华人民共和国民法典》、《中华人民共和国专利法》、《中华人民共和国著作权法》、《中国人民共和国保守国家秘密法》等相关法律法规以及国家《关于加强国家科技计划知识产权管理工作的规定》（国科发政字[2003]9 号）、《关于国家科研计划项目研究成果知识产权管理的若干规定》（国办发[2002]30 号）、科技部《关于加强与科技有关的知识产权保护和管理工作的若干意见》、《科学技术保密规定》等规定执行。

2. 成果管理

本项目执行过程中所产生的成果将按照国家有关规定进行科学管理。在不影响知识产权保护、国家秘密和技术秘密保护的前提下，积极推动项目产生的知识产权和科研成果的转移和运用，加快知识产权的商品化、科研成果的产业化。

3. 合作权益分配

按照国家科技成果相关规定，对项目的研究成果进行权益分配。

（1）合作各单位在申请本课题之前各自获得、拥有的知识产权及相应权益均归各自所有，不因共同申请本课题而改变。

（2）在课题执行过程中，各方应对课题执行过程中产生的科技成果按规定采取知识产权保护措施。

（3）共同完成的科技成果归各方共有，署名顺序按贡献大小由各方商定。

八、需要约定的其他内容

限 500 字以内。

无

九、课题参加人员基本情况表

填表说明： 1.专业技术职称：A、正高级　B、副高级　C、中级　D、初级　E、其他；
2.投入本课题的全时工作时间（人月）是指在课题实施期间该人总共为课题工作的满月度工作量；累计是指课题组所有人员投入人月之和；
3.课题固定研究人员需填写人员明细；
4.是否有工资性收入：Y、是　N、否；
5.人员分类代码：B、课题负责人　C、项目/课题骨干　D、其他研究人员；
6.工作单位：填写单位全称，其中高校要具体填写到所在院系。

序号	姓名	性别	出生日期	证件类型	证件号码	专业技术职称	职务	最高学位	专业	投入本课题的全时工作时间（人月）	人员分类代码	在课题中分担的任务	是否有工资性收入	工作单位
1	佟小光	男	1968-12-26	身份证	130102196812261535	正高级	副院长	博士	神经外科	21	课题负责人	制定临床试验方案，招募临床受试者，开展微创手术进行人工酶电极植入，开展患者监护与随访；开展临床信号检测、致痫灶定位；开展患者深部神经刺激及患者长期管理实验	是	天津市环湖医院
2	尹绍雅	男	1963-09-20	身份证	130402196309200336	正高级	病区主	博士	神经外科	18	课题骨干	制定临床试	是	天津市环湖医院

							任					验方案，招募临床受试者，开展微创手术进行人工酶电极植入，开展患者监护与随访；开展临床信号检测、致痫灶定位；开展患者深部神经刺激及患者长期管理实验		
3	王浩	男	1981-09-30	身份证	120223198109300816	副高级	无	博士	生物医学工程	24	课题骨干	开发临床用闭环神经刺激系统及其接口，提供临床试验样机。开展临床数据分析，参与制定临床方案	是	天津大学医学工程与转化医学研究院
4	朱国行	男	1966-11-21	身份证	33030219661121081X	正高级	无	博士	神经病学	24	课题骨干	制定临床试验方案，招募临床受试者，开展微	是	复旦大学附属华山医院

												创手术进行人工酶电极植入，开展患者监护与随访；开展临床信号检测、致痫灶定位；开展患者深部神经刺激及患者长期管理实验		
5	吴泽翰	男	1990-04-03	身份证	341202199004030914	中级	无	博士	神经外科	30	课题骨干	制定临床试验方案，招募临床受试者，开展微创手术进行人工酶电极植入，开展患者监护与随访；开展临床信号检测、致痫灶定位；开展患者深部神经刺激及患者长期管理实验	是	复旦大学附属华山医院

6	孔祥增	男	1981-12-02	身份证	350822198112026116	正高级	总经理	博士	计算机	18	课题骨干	对受试者脑电信号进行解码和特征值提取，建立癫痫发作监测预测模型	是	厦门隆创思科技有限公司
7	吴玉璋	男	1994-07-24	身份证	370202199407243919	初级	无	硕士	外科学	30	其他研究人员	制定临床试验方案，招募临床受试者，开展微创手术进行人工酶电极植入，开展患者监护与随访；开展临床信号检测、致痫灶定位；开展患者深部神经刺激及患者长期管理实验	否	天津市环湖医院
8	黄立添	男	1985-06-22	身份证	450122198506223011	其他	无	硕士	外科学	30	其他研究人员	制定临床试验方案，招募临床受试者，开展微创手术进行	否	天津市环湖医院

											人工酶电极植入，开展患者监护与随访；开展临床信号检测、致病灶定位；开展患者深部神经刺激及患者长期管理实验		
9	肖朕旭	男	1996-07-10	身份证	330184199607102717	其他	无	学士	临床医学神经病学	30	其他研究人员	制定临床试验方案，招募临床受试者，开展微创手术进行人工酶电极植入，开展患者监护与随访；开展临床信号检测、致病灶定位；开展患者深部神经刺激及患者长期管理实验	否	复旦大学附属华山医院
10	马小茜	女	1998-03-23	身份证	130902199803233222	其他	无	学士	神经病学	30	其他研究	制定临床试	否	复旦大学附属华山医院

										人员	验方案，招募临床受试者，开展微创手术进行人工酶电极植入，开展患者监护与随访；开展临床信号检测、致痫灶定位；开展患者深部神经刺激及患者长期管理实验		
11	陈珂	女	1998-01-07	身份证	41282419980107002X	其他	无	硕士	材料物理与化学	24	其他研究人员	开发临床用闭环神经刺激系统及其接口，提供临床试验样机。开展临床数据分析，参与制定临床方案	否	天津大学医学工程与转化医学研究院
12	裴家晖	女	1996-07-17	身份证	140321199607176046	其他	无	硕士	材料物理与化学	30	其他研究人员	开发临床用闭环神经刺激系统及其接口，提供	否	天津大学理学院

												临床试验样机。开展临床数据分析，参与制定临床方案		
13	赵若荔	女	1997-02-25	身份证	140221199702250026	其他	无	硕士	材料物理与化学	30	其他研究人员	开发临床用闭环神经刺激系统及其接口，提供临床试验样机。开展临床数据分析，参与制定临床方案	否	天津大学理学院
14	闫申	女	1995-06-04	身份证	120225199506040082	其他	无	硕士	机械专业	30	其他研究人员	开发临床用闭环神经刺激系统及其接口，提供临床试验样机。开展临床数据分析，参与制定临床方案	否	天津大学医学工程与转化医学研究院
15	田芳臻	女	1994-12-10	身份证	150202199412104824	其他	无	硕士	智能医学工程	30	其他研究人员	开发临床用闭环神经刺激系统及其接口，提供临床试验样	否	天津大学医学与转化医学研究院

												机。开展临床数据分析，参与制定临床方案		
16	谭可欣	女	1999-11-09	身份证	371502199911095342	其他	无	学士	生物医学工程	30	其他研究人员	开发临床用闭环神经刺激系统及其接口，提供临床试验样机。开展临床数据分析，参与制定临床方案	否	天津大学医学工程与转化医学研究院
17	周苏菲	女	1999-11-09	身份证	332522199911098243	其他	无	学士	生物医学工程	30	其他研究人员	开发临床用闭环神经刺激系统及其接口，提供临床试验样机。开展临床数据分析，参与制定临床方案	否	天津大学医学工程与转化医学研究院
18	朱天宇	女	2000-06-25	身份证	130424200006250042	其他	无	学士	生物医学工程	30	其他研究人员	开发临床用闭环神经刺激系统及其接口，提供临床试验样机。开展临床	否	天津大学医学工程与转化医学研究院

												床数据分析，参与制定临床方案		
19	陈世群	女	1998-09-11	身份证	140429199809118027	其他	无	学士	智能医学工程	30	其他研究人员	开发临床用闭环神经刺激系统及其接口，提供临床试验样机。开展临床数据分析，参与制定临床方案	否	天津大学医学工程与转化医学研究院
20	刘卓洋	男	1991-12-11	身份证	130184199112111032	其他	无	学士	神经外科	30	其他研究人员	开发临床用闭环神经刺激系统及其接口，提供临床试验样机。开展临床数据分析，参与制定临床方案	否	天津市环湖医院
21	李果	男	1998-09-22	身份证	51078119980922711X	其他	无	学士	凝聚态物理	30	其他研究人员	开发临床用闭环神经刺激系统及其接口，提供临床试验样机。开展临床数据分	否	天津大学理学院

												析，参与制定临床方案		
22	刘晓宇	女	1999-05-05	身份证	130725199905050025	其他	无	学士	凝聚态物理	30	其他研究人员	开发临床用闭环神经刺激系统及其接口，提供临床试验样机。开展临床数据分析，参与制定临床方案	否	天津大学理学院
固定研究人员合计										609	/	/	/	/
流动人员或临时聘用人员合计										0	/	/	/	/
累计										609	/	/	/	/

课题预算表

表B1　　课题编号：　2021YFF1200705　　课题名称：　人工酶电极的临床应用研究　　金额单位：万元

序号	预算科目名称	金额
	（1）	（2）
1	一、中央财政专项资金	546.92
2	（一）直接费用	470.92
3	1.设备费	87.00
4	其中：购置设备费	87.00
5	2.业务费	221.72
6	3.劳务费	162.20
7	（二）间接费用	76.00
8	二、其他来源资金	
9	三、合计	546.92

注：1.间接费用无需编制预算说明；2.绩效支出在间接费用中无比例限制。承担单位在统筹安排间接费用时，要处理好合理分摊间接成本和对科研人员激励的关系，绩效支出安排与科研人员在课题工作中的实际贡献挂钩。

设备费——购置/试制设备预算明细表

表B2　课题编号：　2021YFF1200705　　课题名称：　人工酶电极的临床应用研究　　金额单位：万元

填表说明：
1.设备分类：购置、试制；
2.购置设备类型：通用、专用；
3.试制设备不需填列本表（9）列、（10）列、（11）列、（12）列；
4.设备单价的单位为万元/台套，设备数量的单位为台套；
5.单价50万元以下的设备不用填写；
6.本表只填写中央财政资金购置（试制）的设备。

序号	设备名称	设备分类	功能和技术指标	单价	数量	金额	购置或试制单位	安置单位	购置设备类型	主要生产厂家及国别	规格型号	拟开放共享范围
	（1）	(2)	(3)	(4)	(5)	(6)	(7)	(8)	(9)	(10)	(11)	(12)
无记录												
单价50万元以上购置设备合计							/	/	/	/	/	/
单价50万元以上试制设备合计							/	/	/	/	/	/
累计							/	/	/	/	/	/

课题单位经费预算明细表

表B3 课题编号： 2021YFF1200705 课题名称： 人工酶电极的临床应用研究 金额单位：万元

填表说明： 1.单位类型分课题承担单位、课题参与单位；
2.组织机构代码指企事业单位国家标准代码，单位若已三证合一请填写单位统一社会信用代码，无组织机构代码的单位填写“000000000”。

序号	单位名称	组织机构代码-统一社会信用代码		单位类型	任务分工	研究任务负责人	合计	中央财政专项资金		其他来源资金
								小计	其中：间接费用	
	（1）	（2）	（3）	（4）	（5）	（6）	（7）	（8）	（9）	（10）
1	天津市环湖医院	单位组织机构代码	40135431X	课题承担单位	制定临床试验方案，招募临床受试者，开展微创手术进行人工酶电极植入，开展患者监护与随访；开展临床信号检测、致痫灶定位；开展患者深部神经刺激及患者长期管理实验。	佟小光	182.31	182.31	25.33	
2	复旦大学附属华山医院	统一社会信用代码	12100000425006539N	课题参与单位	制定临床试验方案，招募临床受试者，开展微创手术进行人工酶电极植入，开展患者监护与随访；开展临床信号检测、致痫灶定位；开展患者深部神经刺激及患者长期管理实验。	朱国行	136.73	136.73	19.00	
3	天津大学	统一社会信用代码	12100000401359321Q	课题参与单位	开发临床用闭环神经刺激系统及其接口，提供临床试验样机。开展临床数据分析，参与临床方案制定及临床安全性评价。	王浩	136.73	136.73	19.00	
4	厦门隆创思科技有限公司	统一社会信用代码	91350200MA33ALWX0Y	课题参与单位	对受试者脑电信号进行解码和特征值提取，建立癫痫发作监测预测模型。	孔祥增	91.15	91.15	12.67	

课题单位经费预算明细表

表B3 课题编号： 2021YFF1200705 课题名称： 人工酶电极的临床应用研究 金额单位：万元

填表说明： 1.单位类型分课题承担单位、课题参与单位；
2.组织机构代码指企事业单位国家标准代码，单位若已三证合一请填写单位统一社会信用代码，无组织机构代码的单位填写“000000000”。

序号	单位名称	组织机构代码-统一社会信用代码		单位类型	任务分工	研究任务负责人	合计	中央财政专项资金		其他来源资金
								小计	其中：间接费用	
	（1）	（2）	（3）	（4）	（5）	（6）	（7）	（8）	（9）	（10）
累计							546.92	546.92	76.00	

预算说明

一、中央财政资金

预算的编制要坚持任务相关性、政策相符性和经济合理性，实事求是编制提出课题预算。填报时，直接费用应按设备费、业务费、劳务费三个类别填报，每个类别结合科研任务按支出用途进行说明。除50万元以上的设备外，其他费用只提供基本测算说明，不需要提供明细。

1.设备费（是指项目实施过程中购置或试制专用仪器设备，对现有仪器设备进行升级改造，以及租赁外单位仪器设备而发生的费用等。计算类仪器设备和软件工具可在设备费科目编列。填报时，50万元以上的设备详细说明，50万元以下的设备费用分类说明）

（1）购置设备费：

名称	数量	单价/万元	总额/万元	用途
信号采集卡	10	0.70	7.00	人工酶电极临床应用研究中制作数据采集系统
核心工作站	30	2.00	60.00	人工酶电极临床应用研究中分析临床应用中的实验数据
网络数据服务器	10	2.00	20.00	收集分析临床实验中的电信号数据，构建癫痫发作监测预测模型

（2）试制设备费：0

（3）设备改造费：0

（4）设备租赁费：0

2.业务费（是指在项目实施过程中消耗的各种材料、低值易耗品等、发生的测试化验加工、燃料动力、出版文献、信息传播、知识产权事务、会议、差旅、国际合作与交流以及其他与项目实施直接相关的各项费用。编报时，对单笔大额支出、对外委托支出重点说明）

（1）材料费：共计86.4万元（占总经费的15.80%）

单位	名称	数量	单价/万元	总额/万元	用途

环湖医院	患者植入脑电极	69	0.7	48.3	人工酶电极临床应用研究中的参比电极，用于患者脑信号的监测、编码等
	电极线	69	0.02	1.38	人工酶电极临床应用实验中传输电极监测电信号
	总计			49.68	
华山医院	患者植入脑电极	51	0.7	35.7	人工酶电极临床应用研究中的参比电极，用于患者脑信号的监测、编码等
	电极线	51	0.02	1.02	人工酶电极临床应用实验中传输电极监测电信号
	总计			36.72	

具体说明如下：

患者需使用电极进行刺激、监测、编码等，120 例患者，每例患者使用电极 1 个，单价 0.70 万元，共需 125×0.70=84 万元，电极线单价 0.02 万元，共需 120×0.02=2.4 万元。

（2）测试化验加工费 ：共计 87.10 万元（占总经费的 15.93%）

单位	名称	时长次数	单价/万元	总额	用途
环湖医院	脑电图	13 天	0.10/天	1.3	人工酶电极临床应用实验中受试者检测和随访中受试者监护
	核磁共振仪	13 次-69 人	0.05/次	44.85	人工酶电极临床应用实验中受试者检测和随访中受试者监护
	总计			46.15	
华山医院	脑电图	13 天	0.10/天	1.3	人工酶电极临床应用实验中受试者检测和随访中受试者监护
	核磁共振仪	13 次-61 人	0.05/次	39.65	人工酶电极临床应用实验中受试者检测和随访中受试者监护
	总计			40.95	

具体说明如下：测试总周期为 1 年，测试开始前和每月需对患者做脑电图及核磁测试，共 13 次。脑电图每天测试费用为 0.10 万元，共 13×0.1×2=2.6 万元。

为了防止遗漏多准备几次测试每例患者每次的核磁测试费用为 0.05 万元，

共 130×13×0.05=78 万元。

（3）燃料动力费：0

（4）出版/文献/信息传播/知识产权事务费：共计 20.70 万元（占项目总经费 3.78%）

<table>
<tr><td>单位</td><td>项目</td><td>数目</td><td>单价/万元</td><td>总价/万元</td></tr>
<tr><td rowspan="4">天津大学</td><td>国外期刊或国际期刊</td><td>12</td><td>1.00</td><td>12.00</td></tr>
<tr><td>国内专利</td><td>2</td><td>0.80</td><td>1.60</td></tr>
<tr><td colspan="3">查新、文献检索</td><td>4.10</td></tr>
<tr><td colspan="3">资料打印</td><td>3.00</td></tr>
<tr><td colspan="4">合计</td><td>20.70</td></tr>
</table>

预计发表国外期刊或国际会议论文 12 篇，平均每篇论文审稿费、版面费 1.00 万元，总计 12.00 万元。

预计申请国内发明专利 2 项，包括国内发明专利申请书修改、代理、申报等费用，每项平均 0.80 万元，总计 1.60 万元。

进行 11 次文献检索：0.10 万元/次×10 次=1.10 万元。

研究人员和研究生的文献查阅网络使用费，每年需 1.00 万元；三年总计 1.00 万元×3 年=3.00 万元。

用于研究过程中需要支付的复印、打印等费用：

研究生论文印刷费 2.00 元/页（彩页）×200 页×6 本/次×10 次＝2.40 万元；

课题咨询会报告印刷费 2.00 元/页（彩页）×50 页×20 本/次×3 次＝0.60 万元。

（5）会议/差旅/国际合作交流费　：总计 27.52 万元（占总经费的 5.03%）主要用于本项目研究过程中开展科学实验(试验)、科学考察、业务调研、学术交流等所发生的差旅费、市内交通费用。

差旅：在课题进展期间，业务调研/学术交流 5 次，每次 4 天、每次 1 人、人均费用 0.60 万元/次，差旅三年合计 3.00 万元。

课题人员因科研实验、交流等产生的市内交通费，三年合计 3.52 万元。

会议费：为保证项目按计划进行，本课题需要组织技术研讨咨询、评审、审查等会议。按照会议标准（总参会人数不超过 50 人，会期不超过 2 天，会议开支约为 0.05 万元/人天），3 年内计划开展 3 次工作讨论会，每次会期为 2 天。共计：0.05×2 天×30 人×3 次＝9.00 万元。

国际学术会议所需费用：签证费、保险费和会议注册费约 0.40 万元/人，往返交通费按 1.50 万元/人计算。境外住宿费和补贴按 0.1 万元/人天计算，交流 5 天。3 年 5 人次出国参加国际学术会议，共计（0.40 +1.5 +0.1）×5×5 人=12.00 万元。

3. 劳务费（是指在项目实施过程中支付给参与项目的研究生、博士后、访问学者以及项目聘用的研究人员、科研辅助人员、科研（财务）助理等的劳务性费用；支付给临时聘请的咨询专家的费用等。项目聘用人员由单位缴纳的社会保险补助、住房公积金等可纳入劳务费列支。）

（1）劳务/专家咨询费 ：

劳务费共计 150.90 万元（占项目总经费的 27.59%）

**序号**	**人员类型**	**经费标准/万元**	**人数**	**投入时间**	**总额/万元**
环湖医院	患者补助	0.04/天	69	13 天	35.88
	患者保险				17.00
	博士	0.15/月	2	3 年	9.00
	**总计**				**61.88**
华山医院	患者补助	0.04/天	51	13 天	26.52
	患者保险				13.00
	硕士	0.1/天	2	3 年	6.00
	**总计**				**45.52**
天津大学	硕士	0.1/天	7	3 年	21.00
	博士	0.15/月	5	3 年	22.50
	**总计**				**43.50**

具体说明如下：

对患者的监控时长 1 年，其中与课题相关测试时长为 13 天。患者 120 例，每例患者每天补助 0.04 万元，共 120×0.04×13=62.4 万元。

患者在测试过程中，由于可能存在的医疗事故等，需交保险费。按照总实验中心缴纳的费用，本课题承担的患者需交 30 万元。

所需工作人员，包括硕士生和博士生，主要负责对患者的脑电信号的监测，分析整理等工作，每人工作时长 10 月/年，硕士每月发放劳务费 0.1 万元，博士每月发放劳务费 0.15 万元，共 0.10×3×10×9+0.15×3×10×7=58.50 万元。

（2）专家咨询费共计 11.30 万元（占总经费的 2.07%）

以会议形式组织的咨询。支出明细如下：

序号	专家类型	会议名称	次数	天数	人数	标准/万元	总额/万元
1	正高级	课题启动会	1	3	5	0.10	1.50
2	正高级	课题中期检查会	1	1	6	0.60	0.60
3	正高级	课题年度检查会	3	2	7	0.10	4.20
4	正高级	课题验收会	1	2	10	0.10	2.00
5	正高级	课题咨询会	3	2	5	0.10	3.00
**总计**							**11.30**

项目开始初期需召开项目工作布置会 1 次，聘请领域内的 5 名高级专家组成咨询委员会，共计 1.00 万元；

项目第二年需要召开一次中期检查会议，聘请领域内的 6 名高级专家组成咨询委员会，共计 0.60 万元；

项目第三年需要召开一次课题验收会议，聘请领域内的 10 名高级专家组成咨询委员会，共计 2.00 万元；

项目每年需要召开一次课题年度检查会议，聘请领域内的 7 名高级专家组成咨询委员会，共计 4.20 万元；

课题召开学术研讨会专家咨询费，根据项目的研究内容及课题研究过程中出现的问题聘请专家全程对课题进展进行咨询，以每年咨询 1 次，每次聘请该领域内的 5 名高级专家，每次为期 2 天。则三年总计为：3.00 万元。

以现场指导形式专家咨询费；

拟聘请人工酶电极、神经编解码领域内经验丰富的 5 名专业技术人员分多次现场指导，以三年现场指导咨询 5 次，每次 3 人，每次 2 天，则 0.10 万元/人天×3 人×2 天×5 次=3.00 万元。

二、其他来源资金

对其他来源资金主要用途、支出预算做简要说明。

无

十一、相关附件

1. 乙方与参加单位有关协议（须加盖乙方与参加单位公章、法人签字签章；协议文件须扫描上传。如无参加单位，则不填）；

2. 申报指南规定的其他附件。

课题合作实施协议：

国家重点研发计划

“生物与信息融合(BT与IT融合)”重点专项

“新一代高相容性生物植入电极设计与应用”项目

课题五“人工酶电极的临床应用研究”

合作实施协议

依据《中华人民共和国科技进步法》、《中华人民共和国民法典》、《中华人民共和国著作权法》、《中华人民共和国专利法》、《中华人民共和国促进科技成果转化法》、《关于改进加强中央财政科研项目和资金管理的若干意见》等法律和管理办法，经协商一致，各方同意就国家重点研发计划“生物与信息融合(BT与IT融合)”重点专项，“新一代高相容性生物植入电极设计与应用”项目，课题五“人工酶电极的临床应用研究”签订合作实施协议。

第1条 联合体组成

天津市环湖医院作为该课题的承担单位，复旦大学附属华山医院，天津大学，厦门隆创思科技有限公司作为该课题的参加单位（以下简称"合作方"）。

第2条 联合体分工

联合体各方的任务分工如下：

课题五、人工酶电极的临床应用研究

本课题由天津市环湖医院牵头组织，复旦大学附属华山医院，天

津大学,厦门隆创思科技有限公司参加实施。按照课题目标和研究内容要求，本课题具体任务及经费分配如下：

承担单位：天津市环湖医院

参加单位：复旦大学附属华山医院,天津大学,厦门隆创思科技有限公司

经费安排：占项目总经费（2363.01 万元）的 23.15%

具体任务：结构性癫痫患者，植入人工酶电极进行脑电信号检测；开展致痫灶定位研究（>120 例）；建立癫痫发作监测、预测模型。建立深部神经刺激治疗方案，开展人工酶电极闭环反应性神经刺激。开展人工酶电极对临床患者的长期管理，包括临床用药方案调整，脑区神经功能评价等。

考核指标：实现癫痫患者致痫灶定位，深部神经刺激及长期管理，建立癫痫发作监测、预测模型，开展患者长期管理。受试者数目 120 例以上；信号采集时间跨度 1 年以上；开发癫痫发作监测预测模型 2 种以上，预测准确性大于 99%；发表高水平论文 6 篇以上，提供论文检索证明；申请专利 2 项以上，提供专利初步审查合格通知书。

具体任务分工和考核指标分配如下：

单位名称	任务分工	考核指标
天津市环湖医院	制定临床试验方案，招募临床受试者，开展微创手术进行人工酶电极植入，开展患者监护与随访；开展临床信号检测、致痫灶定位；开展患者深部神经刺激及患者长期管理实验	受试者数目 60 例以上；开展致痫灶定位、深部神经刺激的临床试验及患者长期管理；发表高水平论文 1 篇以上
复旦大学附属华山医院	招募临床受试者，开展微	受试者数目 60 例以上；

	创手术进行人工酶电极植入，开展患者监护与随访；开展临床信号检测、致痫灶定位；开展患者深部神经刺激及患者长期管理实验	开展致痫灶定位、深部神经刺激的临床试验及患者长期管理；发表高水平论文1篇以上
天津大学	开发临床用闭环神经刺激系统及接口，提供临床试验样机。开展临床数据分析，参与临床方案制定及临床安全性评价	人工酶电极长期应用信号采集时间跨度 1 年以上；发表高水平论文2篇以上，申请专利1项以上
厦门隆创思科技有限公司	对受试者脑电信号进行解码和特征值提取，建立癫痫发作监测预测模型	癫痫发作检测预测模型开发癫痫发作监测预测模型 2 种以上，预测准确性大于 99%；发表高水平论文 2 篇以上，申请专利1项以上。

第3条 课题经费分配及承担

各方就该课题的国拨专项经费在“新一代高相容性生物植入电极设计与应用”项目中的分配比例，及自筹部分承诺如下：

课题名称	单位名称	国拨比例	自筹金额（万元）
课题五：人工酶电极的临床应用研究	天津市环湖医院	7.72%	0
	天津大学	5.79%	0
	复旦大学附属华山医院	5.79%	0
	厦门隆创思科技有限公司	3.86%	0

项目经费额度以科技部下达的任务（合同）书为准。

第4条 知识产权管理

4.1 承担单位与参加单位在实施本课题之前各自所获得的知识产权及相应权益均归各自所有，不因共同实施本课题而改变。

4.2 因实施课题的需要，各自向对方提供的未公开的、或在提供之前已告知不能向第三方提供的与本课题相关的技术资料、数据等所有信息，包括但不限于各自所有或合法拥有的任何计算机程序、代码、算法、公式、过程、观念、图标、照片、制图、设计、产品、样品、发明创造（包括发明、实用新型和外观设计，无论是否获得专利）、技术秘密、版权、商标、产品研发计划、预测、策略、规范、实际或潜在商业活动的信息，客户与供应商名单、财务事项、市场营销计划等技术、商务信息等。未经提供方同意，不得提供给第三方。该条款长期有效。

4.3 因实施本课题的需要，各自向对方提供的相关信息，不构成向对方授予任何关于专利权、著作权、商标权等知识产权的许可行为。

第 5 条 补充协议或争议解决办法

5.1 在课题实施过程中发生争议，联合体各方应当协商解决。各方不愿协商、调解解决或者协商、调解不成的，商定申请由天津仲裁委员会仲裁。

第 6 条 有效期

6.1 本协议一式 6 份，协议签订双方各执 2 份，2 份上交主管部门，具有同等法律效力。

6.2 本协议自课题批准立项之日起生效，有效期至课题结项之日。

以下无正文，转签章页。

本页无正文，为签章页

（课题/任务牵头单位签章页）

课题承担单位（公章）：天津市环湖医院

课题参加单位（公章）：

法定代表人签字：葛乐

法定代表人签字：金东寒印

课题负责人（签字）

子课题/任务负责人（签字）王浩

2021 年 12 月 8 日

2021 年 11 月 19 日

（此件仅用于国家重点研发计划"生物与信息融合(BT 与 IT 融合)"重点专项"新一代高相容性生物植入电极设计与应用" 课题五 "人工酶电极的临床应用研究" 课题合作实施）

以下空白

本页无正文，为签章页

（课题/任务牵头单位签章页）

课题承担单位（公章）：

法定代表人签字：

课题负责人（签字）

2021年12月8日

课题参加单位（公章）

法定代表人签字：

子课题/任务负责人（签字）

2021年12月1日

（此件仅用于国家重点研发计划"生物与信息融合(BT与IT融合)"重点专项"新一代高相容性生物植入电极设计与应用" 课题五 "人工酶电极的临床应用研究" 课题合作实施）

以下空白

本页无正文，为签章页

（课题/任务牵头单位签章页）

课题承担单位（公章）：天津市环湖医院

法定代表人签字：葛乐

课题负责人（签字）

2021 年 12月 8日

课题参加单位（公章）：厦门[illegible]思科技有限公司 3502061004447

法定代表人签字：

子课题/任务负责人（签字）

2021 年 11月 18 日

（此件仅用于国家重点研发计划"生物与信息融合(BT 与 IT 融合)"重点专项"新一代高相容性生物植入电极设计与应用" 课题五 "人工酶电极的临床应用研究" 课题合作实施）

以下空白

数据汇交承诺书：

附件:

科学数据汇交承诺书

对本项目产生的样本信息、临床病例信息、队列大数据、实验数据等科学数据做出以下承诺：

1.承诺本项目汇交的数据真实、准确、完整、有效，无学术不端和学术失范行为，并承担因数据不真实等问题带来的一切后果和法律责任。

2.承诺将本项目产生的样本信息、临床病例信息、队列大数据、实验数据等科学数据无条件汇交到科技部指定平台。

3.承诺及时组织汇交科学数据给项目承担单位及数据中心。

4.本承诺书自签字盖章之日起生效。

项目/课题负责人（签章）：[illegible]

项目/课题牵头单位（签章）：天津市环湖医院

2021 年 12 月 8 日

人类遗传资源承诺书：

附件：

人类遗传资源管理和伦理审查承诺书

本人承诺在项目实施过程中，涉及人的生物医学研究的，严格按照《涉及人的生物医学研究伦理审查办法》等规定执行；涉及人体研究需按照规定通过伦理审查并签署知情同意书。如涉及人类遗传资源；严格按照《中华人民共和国人类遗传资源管理条例》等法规执行；涉及生物技术研究的，严格遵守《生物技术研究开发安全管理办法》等规章；涉及病原微生物研究的，严格遵守《病原微生物实验室安全管理条例》等法规；涉及实验动物和动物实验的，严格遵守国家实验动物管理的法律、法规、技术标准及有关规定，使用合格实验动物，在合格设施内进行动物实验，保证实验过程合法，实验结果真实、有效，并通过实验动物福利和伦理审查。承诺相关实验接受有关管理机构监督检查。

项目/课题负责人（签章）：[illegible]

项目/课题牵头单位（签章）：天津市环湖医院

2021 年 12月 8日

参加项目的企业营业执照：厦门隆创思

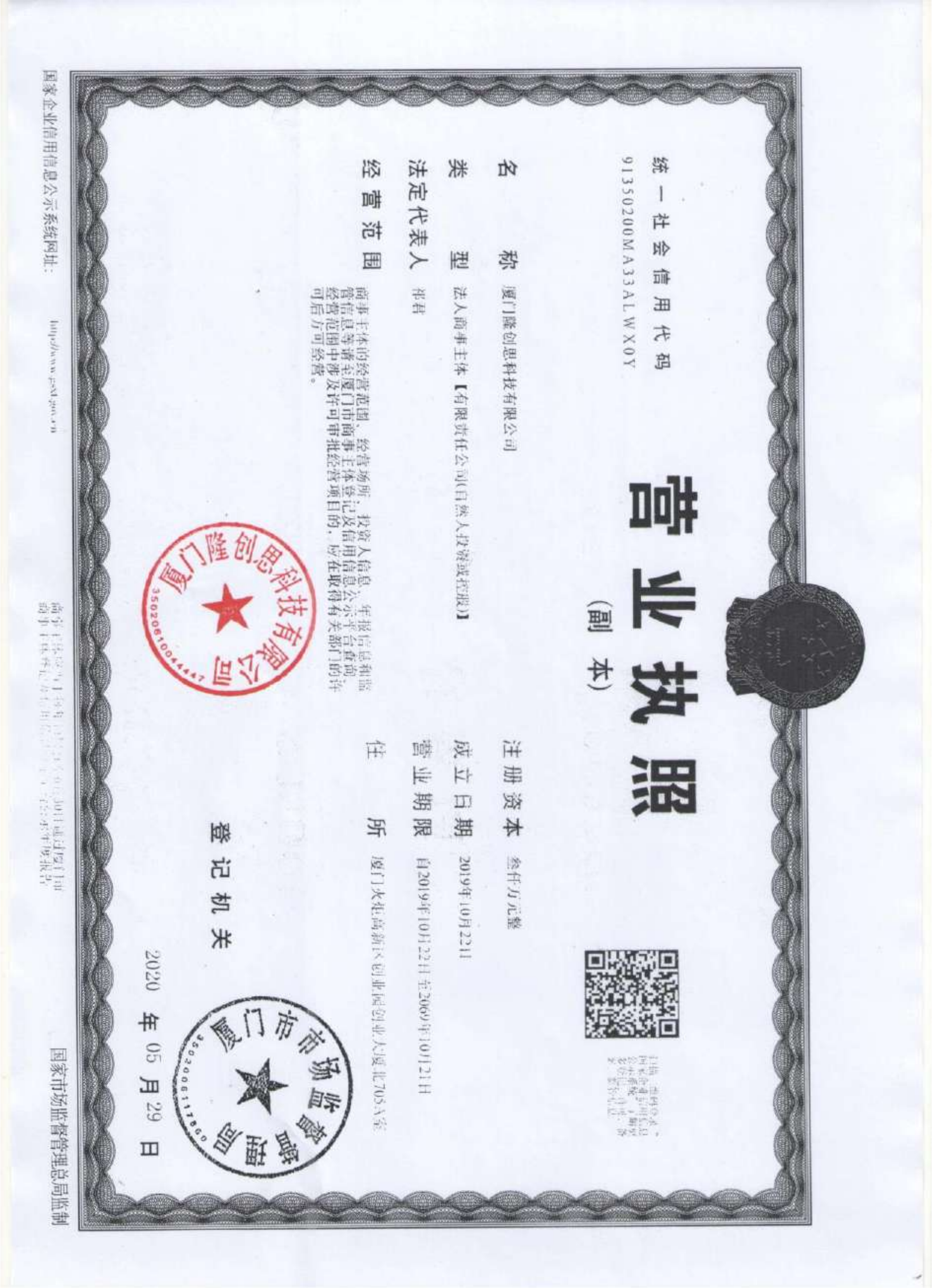

统一社会信用代码
91350200MA33ALWX0Y

营业执照

（副 本）

名称 厦门隆创思科技有限公司

类型 法人商事主体【有限责任公司(自然人投资或控股)】

法定代表人 祁君

经营范围 商事主体的经营范围、经营场所、投资人信息、年报信息和监管信息等请至厦门市商事主体登记及信用信息公示平台查询。经营范围中涉及许可审批经营项目的，应在取得有关部门的许可后方可经营。

注册资本 叁仟万元整

成立日期 2019年10月22日

营业期限 自2019年10月22日至2069年10月21日

住所 厦门火炬高新区创业园创业大厦北705A室

登记机关

2020 年 05 月 29 日

国家企业信用信息公示系统网址： http://www.gsxt.gov.cn

商事主体应当于每年[illegible]30日通过厦门市商事主体登记及信用信息[illegible]公示平台报送年度报告

国家市场监督管理总局监制

临床试验伦理审批：天津市环湖医院

天津市环湖医院伦理委员会

伦理审查批件

批件号	（津环）伦审第（2021-057）号				
项目名称	一项新一代高相容性生物植入电极功能与安全性评价研究项目				
项目来源	天津市环湖医院				
研究单位	天津市环湖医院		承担科室	神经外科	
主要研究者	佟小光		职称	主任医师	
审查类别		审查方式		审查日期	
初始审查		会议审查		2021年6月29日	
审查地点	天津市环湖医院伦理委员会会议室				
审查委员	王新平、张延铭、张金玲、韩彤、李毅、李明宇、闫贵明				
批准文件	会审批文件：<br>1.临床研究方案<br>2.知情同意书				

审查意见：同意
根据国家《涉及人的生物医学研究伦理审查办法》、《药物临床试验质量管理规范》、《药物临床试验伦理审查工作指导原则》、《医疗器械临床试验质量管理规范》、《体外诊断试剂临床研究技术指导原则》、WMA《赫尔辛基宣言》和CIOMS《人体生物医学研究国际道德指南》的伦理原则，经本伦理委员会审查，同意按所批准的文件开展本项研究。<br>请遵循GCP原则、遵循伦理委员会批准的方案开展临床研究，保护受试者的健康与权利。

年度/定期跟踪审查频率	一年		
有效期	该批件有效期一年（自批准之日起），如试验逾期未实施需提出延长有效期申请。		
联系人	李诚	联系电话	022-59065833
主任委员签字	王莉莉		
伦理委员会	天津市环湖医院医学伦理委员会（盖章）		
日期	2021年6月30日		

标准操作规程/Standard Operating Procedures 第2页

临床试验伦理审批：复旦大学附属华山医院

医院伦理审查委员会声明

我单位朱国行同志参与申报的国家重点研发计划“新一代高相容性生物植入电极设计与应用”项目，当中涉及人的临床研究。我们在此承诺，如果本项目正式立项，将按照国家各部委涉及人的生物医学研究相关管理规范对其进行伦理审查，待通过伦理审查后方进入临床研究，并在我单位伦理审查委员会跟踪审查中开展相关研究工作，确保研究符合国家各部委的相关规定，维护患者权益。

特此声明。

复旦大学附属华山医院医院伦理委员会（盖章）

2021年6月24日

知情同意书——知情告知页

亲爱的患者：

您的医生已经确诊您患有 局灶性癫痫 疾病，我们邀请您参加一项 新一代高相容性生物植入电极功能与安全性评价 研究项目，该项目为 国家重点研发计划 项目，本研究方案已经得到 天津市环湖医院伦理委员会 审核批准，同意进行临床研究。

我们将邀请您参加此项应用基础研究，在您决定参加这项研究之前，请尽可能仔细阅读以下内容，它可以帮助您了解该项研究以及为何要进行这项研究，研究的程序和期限，参加研究后可能给您带来的益处、风险和不适。如果您愿意,您也可以和您的亲属、朋友一起讨论,或者请您的医生给予解释，帮助您做出决定 。

一、 研究背景和研究目的

1.疾病负担和治疗现状

癫痫是影响所有年龄人群的一种脑部慢性非传染性疾病。全世界有大约5000万癫痫患者，使之成为全球范围内最常见的神经系统疾病之一，近80%生活在中低收入国家，占全球疾病总负担 0.6%。其中，我国有高达900万以上人群受累，并以每年60万例的速度持续递增，年经济负担超过200亿人民币。与普通人群相比，癫痫患者的过早死亡风险要高出3倍。癫痫患者中约有三分之一单纯药物治疗效果差，需要手术切除致病大脑组织，其中部分病人因发病部位关联关键生命获得或重要人体功能而不适应手术。

2.研究背景与研究目的

脑深部电刺激是利用立体定向的方法在脑内特定的核团或脑区植入刺激电极并通过特定频率的电刺激调控相关核团或脑区的功能达到改善疾病症状的目的与传统毁损性手术相比具有微创、可逆、可调节的特点。近年来深部电刺激已经被用于癫痫，帕金森症，脊髓损伤等各种神经系统疾病的治疗上，并且取得了良好的疗效。同时植入电极还可采集患者脑电信号，监测发病情况开展患者用药调整，进行脑区功能评价开展患者长期管理，对于癫痫患者具有重要治疗和监测作用，可显著提高治疗效果，降低患者负担。但目前植入电极仍存在电极材料与组织界面生物相容性差，易致炎症反应，进而导致灵敏度降低等问题。国家重点研发计划“生

物与信息融合（BT与IT融合）”重点专项，项目“新一代高相容性生物植入电极设计与应用”拟应用人工酶对植入电极进行表面重构，开发新一代人工酶电极，提高神经电极的检测灵敏度，并实现在体神经信号的长期采集，提高植入电极的临床实用性。本研究即在此项目下通过结构性癫痫患者植入实验，评价新一代高相容性生物植入电极功能与安全性。

3. 研究参加单位和预计纳入参试者例数

本研究将在 天津市环湖医院 进行，预计将有120名患者自愿参加,研究期限3年。天津市环湖医院伦理委员会 已经审批此项研究是遵从赫尔辛基宣言原则,符合医疗道德的。

二、哪些人不宜参加研究

（1）手术部位感染或严重污染；

（2）严重神经精神障碍或其他癫痫手术禁忌症；

（2）装有心脏起搏器的患者；

（3）研究人员认为其他原因不适合临床试验者；

（4）正参加其它临床试验的患者。

三、如果参加研究将需要做什么？

1.在您入选研究前，医生将询问、记录您的病史，并进行检查。如您是合格的纳入者，您可自愿参加研究，签署知情同意书。如您不愿参加研究，我们将按您的意愿施治。

2.若您自愿参加研究，将按以下步骤进行：

简单叙述患者分配流程、各治疗方案（药物：剂量、疗程、使用说明和注意事项、药物生产厂家和批号；采用的治疗和诊断仪器：生产厂家、使用说明和注意事项） 患者到医院进行检查和随访的时间、次数、注意事项。

3. 需要您配合的其他事项

您必须按医生和您约定的随访时间带着病历、个人治疗日记卡等来医院就诊医生可能通过电话、登门的方式了解您的情况。您的随访非常重要，因为医生将判断您接受的治疗是否真正起作用，并及时指导您。您必须按医生指导用药，并请您及时、客观地填写您的服药记录。您在每次随访时都必须归还未用完的药物及其包装，并将正在服用的其它药物带来，包括您有其

它合并疾病须继续服用的药物。在研究期间您不能使用治疗癫痫的其它药物。如您需要进行其它治疗，请事先与您的医生取得联系。

四、参加研究可能的受益

社会将从这项研究中受益，此种受益包括人类对于癫痫发生、发展的认识以及用于癫痫诊断治疗相应医疗器械的获得。

尽管已经有证据提示__通过植入电极开展癫痫患者长期管理和深部神经刺激治疗__有满意的疗效，但这并不能保证对您肯定有效。本研究所采用的__新一代高相容性植入电极__也不是治疗__癫痫__的唯一的方法。如本研究对您的病情无效，您可以向医生询问有可能获得的替代治疗方法。

五、参加研究可能的风险、不良反应、不适、不方便

这项研究可能的不良反应为过敏反应，如发生严重过敏反应医生会及时停止试验，并对过敏反应进行及时治疗，对您的疾病开展替代治疗。

如果在研究期间您出现任何不适，或病情发生新的变化，或任何意外情况，不管是否与研究有关，均应及时通知您的医生，他/她将对此作出判断并给与适当的医疗处理。

您在研究期间需要按时到医院随访，做一些检查，这些占用您的一些时间，也可能给您造成麻烦或带来不方便。

六、有关费用

本项研究不增加您的任何费用，本研究所采用的植入电极及植入手术费用，与本研究相关的检查费用将为您减免。对于您同时合并的其他疾病所需的治疗和检查，将不在免费的范围之内。

七、个人信息的保密

您的医疗记录（研究病历/CRF、化验单等）将完整地保存在您所就诊的医院。医生会将化验和其它检查结果记录在您的病历上。研究者、伦理委员会和药品监督管理部门将被允许

查阅您的医疗记录。任何有关本项研究结果的公开报告将不会披露您的个人身份。我们将在法律允许的范围内，尽一切努力保护您个人医疗资料的隐私。按照医学研究伦理，除了个人隐私信息外，试验数据将可供公众查询和共享，查询和共享将只限于基于网络的电子数据库，保证不会泄漏任何个人隐私信息。

八、怎样获得更多的信息？

您可以在任何时间提出有关本项研究的任何问题，并得到相应的解答。如果在研究过程中有任何重要的新信息，可能影响您继续参加研究的意愿时，您的医生将会及时通知您。

九、可以自愿选择参加研究和中途退出研究

是否参加研究完全取决于您的意愿。您可以拒绝参加此项研究，或在研究过程中的任何时间退出本研究，这都不会影响您和医生间的关系，都不会影响对您的医疗或有其他方面利益的损失。出于对您的最大利益考虑，医生或研究者可能会在研究过程中随时中止您继续参加本项研究。如果您因为任何原因从研究中退出，您可能被询问有关您使用试验药物的情况。如果医生认为需要，您也可能被要求进行实验室检查和体格检查。

十、现在该做什么？

是否参加本项研究由您自己（和您的家人）决定。在您做出参加研究的决定前，请尽可能向你的医生询问有关问题。感谢您阅读以上材料。如果您决定参加本项研究，请告诉您的医生，他/她会为您安排一切有关研究的事务。请您保留这份资料。

知情同意书——同意签字页

研究项目名称： 新一代高相容性生物植入电极功能与安全性评价

项目负责人： 佟小光

项目执行单位： 天津市环湖医院

同意声明：

我已经阅读了上述有关本研究的介绍，而且有机会就此与医生讨论并提出问题，我提出的所有问题都得到了满意的答复。我知道参加研究可能的风险和受益，我知晓参加研究是自愿的，我确认已有充足时间对此进行考虑，而且明白：

- 我可以随时向医生咨询更多的信息。
- 我可以随时退出本研究，而不会受到歧视或报复，医疗待遇与权益不会受到影响。

我同样清楚，如果我中途退出研究，特别是由于试验的原因使我退出研究时，我若将我的病情变化告诉医生，完成相应的体格检查和理化检查，这将对整个研究十分有利。

如果因病情变化我需要采取任何其他的治疗，我会在事先征求医生的意见，或在事后如实告诉医生。

我同意研究者、伦理委员会或管理部门查阅我的病历资料

我同意除本研究以外的其他研究利用我的医疗记录和组织标本

我将获得一份经过签名并注明日期的知情同意书副本

最后，我决定同意参加本项研究，并遵从医嘱。

受试者签名：________________ 日期：________________

受试者联系电话：________________________________

医生声明：

我确认已向患者解释了本实验的详细情况，包括其权利以及可能的受益和风险，并给其一份签署过的知情同意书副本。

研究者签名：________________ 日期：________________

研究者工作电话：________________________________

临床试验知情同意书：华山医院

“药物/医疗器械临床研究”受试者须知模版

（括号内斜体字部分需根据课题不同情况自行填写）

方案名称：新一代高相容性生物植入电极功能与安全性评价

方案编号：　KY2021-

方案版本号：01，2020年6月22日

知情同意书版本号：01，2020年6月22日

研究机构：复旦大学附属华山医院

主要研究者（负责研究医师）：朱国行

尊敬的受试者：

您将被邀请参加一项临床研究。本须知提供给您一些信息以帮助您决定是否参加此项临床研究。请您仔细阅读，如有任何疑问请向负责该项研究的研究者提出。我们将和您或您的家人进行详细沟通，向您介绍该项研究的有关情况，如果您同意参与这项研究，也请您提供与疾病有关的情况，包括发病过程、家族史、以前就诊情况及曾经做过一些检查结果等，我们将对您进行编号，建立病历档案。

您参加本项研究是自愿的。本次研究已通过本研究机构伦理审查委员会审查。

1.研究概况：癫痫是影响所有年龄人群的一种脑部慢性非传染性疾病。全世界有大约5000万癫痫患者，使之成为全球范围内最常见的神经系统疾病之一，近80%生活在中低收入国家，占全球疾病总负担0.6%。其中，我国有高达900万以上人群受累，并以每年60万例的速度持续递增，年经济负担超过200亿人民币。与普通人群相比，癫痫患者的过早死亡风险要高出3倍。癫痫患者中约有三分之一单纯药物治疗效果差，需要手术切除致病大脑组织，其中部分病人因发病部位关联关键生命获得或重要人体功能而不适应手术。脑深部电刺激是利用立体定向的方法在脑内特定的核团或脑区植入刺激电极并通过特定频率的电刺激调控相关核团或脑区的功能达到改善疾病症状的目的与传统毁损性手术相比具有微创、可逆、可调节的特点。近年来深部电刺激已经被用于癫痫，帕金森症，脊髓损伤等各种神经系统疾病的治疗上，并且取得了良好的疗效。同时植入电极还可采集患者脑电信号，监测发病情况开展患者用药调整，进行脑区功能评价开展患者长期管理，对于癫痫患者具有重要治疗和监测作用，可显著提高治疗效果，降低患者负担。但目前植入电极仍存在电极材料与组织界面生物相容性差，易致炎症反应影响界面输运，进而导致灵敏度降低等痛点和难点问题。国家重点研发计划“生物与信息融合（BT与IT融合）”重点专项，项目“新一代高相容性生物植入电极设计与应用”通过人工智能和量子力学方法辅助设计

团簇级人工酶对植入电极进行原子尺度的设计，提高神经电极的检测灵敏度，并实现在体神经信号的长期采集，大幅度提高电极的生物相容性，提高植入电极的临床实用性。本研究即在此项目下通过结构性癫痫患者植入实验，评价新一代高相容性生物植入电极功能与安全性。

2.研究目的：通过结构性癫痫患者植入实验，评价新一代高相容性生物植入电极功能与安全性。

3.研究过程：本研究将在复旦大学附属华山医院进行，预计将有 120 名患者自愿参加,研究期限 3 年。复旦大学附属华山医院伦理委员会 已经审批此项研究是遵从赫尔辛基宣言原则,符合医疗道德的。本研究采用随机分组的方法，将参加试验的患者随机分到对照组或试验组。由于临床诊断和治疗需要，您要进行脑电极植入的外科手术，术后从第 7 天起开展脑电信号采集，建立癫痫脑电信号监测预测模型。通过对癫痫信号的监测制定神经刺激治疗方案，开展深部神经刺激对癫痫的治疗。通过对照组和试验组的神经炎症反应，对比市售电极与人工酶电极对脑组织排异反应的抑制率差异，评价人工酶电极的生物相容性。通过对脑电信号检测的灵敏度与精密度对比评价市售电极与人工酶电极的灵敏度与稳定性。在实验过程中医生会对您进行体格检查，脑电图、脑核磁检查，通过脑电极进行脑电检测根据您的病情进行脑电刺激等。

4. 风险与不适：对于您来说，与我们进行沟通、交谈可能会有些心理不适。您在研究期间需要按时到医院随访，做一些检查，这些占用您的一些时间，也可能给您造成麻烦或带来不方便。这项研究可能的不良反应为过敏反应，如发生严重过敏反应医生会及时停止试验，并对过敏反应进行及时治疗，对您的疾病开展替代治疗。如果在研究期间您出现任何不适，或病情发生新的变化，或任何意外情况，不管是否与研究有关，均应及时通知您的医生，他/她将对此作出判断并给与适当的医疗处理。

5. 潜在获益：通过对您的脑电信号进行检测或研究可能有助于对您的疾病作出诊断，也可能为您的治疗提供必要的建议和治疗方案。社会将从这项研究中受益，此种受益包括人类对于癫痫发生、发展的认识以及用于癫痫诊断治疗相应医疗器械的获得。尽管已经有证据提示 通过植入电极开展癫痫患者长期管理和深部神经刺激治疗 有满意的疗效，但这并不能保证对您肯定有效。本研究所采用的 新一代高相容性植入电极 也不是治疗 癫痫 的唯一的方法。如本研究对您的病情无效，您可以向医生询问有可能获得的替代治疗方法。

6. 费用：本项研究不增加您的任何费用，本研究所采用的植入电极及植入手术费用，与本研究相关的检查费用将为您减免。对于您同时合并的其他疾病所需的治疗和检查，将不在免费的范围之内。

7. 补偿：本项研究将按国家规定为您提供与研究活动相关的交通费和误餐费。

8. 本次研究之外的备选疗法：如因各种原因导致本次研究中断，我们将为您采用备选疗法，如药物控制或手术毁损治疗等。

9. 如果您因参与这项研究而受到伤害：如发生与临床研究相关的损害时，您可以获得免费治疗和 / 或相应的补偿。

10.受试者职责：作为研究受试者，您有以下职责：提供有关自身病史和当前身体状况的真实情况；告诉研究医生自己在本次研究期间所出现的任何问题；不得服用受限制的药物、食物等；告诉研究医生自己在最近是否曾参与其他研究，或目前正参与其他研究；配合医生完成各项检查、治疗、随访等。

11. 隐私问题：如果您决定参加本项研究，您参加研究及在研究中的个人资料均属保密。负责研究医师及其他研究人员将使用您的医疗信息进行研究。这些信息可能包括您的姓名、地址、电话号码、病史及在您研究来访时得到的信息。您的档案将保存在有锁的档案柜中，仅供研究人员查阅。研究中会用编号来标识您的研究信息和所采集的生物样本。只有研究者和研究小组成员可查询编号。为确保研究按照规定进行，必要时，研究申办者，政府管理部门或伦理审查委员会的成员按规定可以在研究单位查阅您的个人资料。这项研究结果发表时，将不会披露您个人的任何资料。

12. 生物样本和信息的处理：您的生物样本和信息仅会提供给本项目研究人员，在特定情况下也会提供给上级主管单位；试验结束后您的生物样本和信息将按规定保存在复旦大学附属华山医院。

13. 研究的资金来源，与医疗机构/研究者可能的利益冲突：本次研究经费来源为国家重点研发计划“生物与信息融合（BT与IT融合）”重点专项，“新一代高相容性生物植入电极设计与应用”项目。

14. 其他：

您可以选择不参加本项研究，或者在任何时候通知研究者后退出而不会遭到歧视或报复，您的任何医疗待遇与权益不会因此而受到影响。

如果您需要其它治疗，或者您没有遵守研究计划，或者发生了与研究相关的损伤或者有任何其它原因，研究医师可以终止您继续参与本项研究。

您可随时了解与本研究有关的信息资料和研究进展，若发生与本研究相关的安全性新信息，我们也会及时通知您。如果您有与本研究有关的问题，或您在研究过程中发生了任何不适与损伤，或有关于本项研究参加者权益方面的问题您可以通过 021-52888472 （*手机号码或保证24小时联系畅通的座机号码*）与 朱国行 （*研究者或有关人员姓名*）联系。

如果您对参与本研究的权益和健康有任何问题或诉求，您可以联系本机构伦理委员会，联系电话：52888045。

知情同意书签字页

我已经阅读了本知情同意书。

我有机会提问而且所有问题均已得到解答。

我理解参加本项研究是自愿的。

我可以选择不参加本项研究，或者在任何时候通知研究者后退出而不会遭到歧视或报复，我的任何医疗待遇与权益不会因此而受到影响。

如果我需要其它治疗，或者我没有遵守研究计划，或者发生了与研究相关的损伤或者有任何其它原因，研究医师可以终止我继续参与本项研究。

我将收到一份签过字的“知情同意书”副本。

受试者姓名：________________________

受试者签名：________________________

日期：______年_______月_______日

监护人姓名：________________________

监护人签名：________________________

日期：______年______月______日 *（适用于受试者无认知或行为能力或为未成年人）*

见证人姓名：________________________

见证人签名：________________________

日期：______年_______月_______日 *（适用于受试者或监护人无阅读能力，或其他需要独立于研究之外的见证人的情形）*

我已准确地将这份文件告知受试者，要求他/她认真阅读了这份知情同意书，对所提出的问题或疑问认真解答。

研究者姓名：________________________

研究者签名：________________________

日期：______年_______月_______日

（注：若受试者或监护人无阅读能力，需公正的见证人签名（公正见证人，指与临床研究无关，不受临床研究相关人员不公正影响的个人，在受试者或者其监护人无阅读能力时，作为公正的见证人，阅读知情同意书和其他书面资料，并见证知情同意。）；若受试者无行为或认知能力，则需获得监护人的书面知情同意；若受试者是未成年人，应获得监护人的书面知情同意，同时应获得8-18岁受试者的书面知情同意和8岁以下受试者的口头知情同意）

课题负责人诚信承诺书：佟小光

国家科技计划项目申报诚信承诺书

（申请人部分）

本人根据国家重点研发计划项目申报指南的要求自愿提交项目（课题）申报书，**在此郑重承诺：**严格遵守《关于进一步加强科研诚信建设的若干意见》、《关于进一步弘扬科学家精神 加强作风和学风建设的意见》、《科技部 自然科学基金委关于进一步压实国家科技计划（专项、基金等）任务承担单位科研作风学风和科研诚信主体责任的通知》、《科学技术活动评审工作中请托行为处理规定（试行）》等有关规定，杜绝《科学技术活动违规行为处理暂行规定》所列违规行为，所申报材料和相关内容真实有效，不存在违背科研诚信要求的行为；已按要求落实了科研作风学风和科研诚信主体责任；不以任何形式实施请托行为，申报材料符合《中华人民共和国保守国家秘密法》和《科学技术保密规定》等相关法律法规；在参与国家科技计划项目申报、评审和实施全过程中，恪守职业规范和科学道德，遵守评审规则和工作纪律，杜绝以下行为：

（一）抄袭、剽窃他人科研成果或者伪造、篡改研究数据、研究结论；

（二）购买、代写、代投论文，虚构同行评议专家及评议意见；

（三）违反论文署名规范，擅自标注或虚假标注获得科技计划等资助；

（四）违反科研伦理规范；

（五）弄虚作假，骗取科技计划项目、科研经费以及奖励、荣誉等；

（六）在正式申报书中以高指标通过评审，在任务书签订时故意篡改降低任务书中相应指标；

（七）以任何形式探听尚未公布的评审专家名单及其他评审过程中的保密信息；

（八）本人或委托他人通过各种方式及各种途径联系有关专家进行请托、游说，违规到评审会议驻地游说评审专家和工作人员、询问评审或尚未正式向社会公布的信息等干扰评审或可能影响评审公正性的活动；

（九）向评审工作人员、评审专家等提供任何形式的礼品、礼金、有价证券、支付凭证、商业预付卡、电子红包，或提供宴请、旅游、娱乐健身等任何可能影响评审公正性的活动；

（十）其它违反财经纪律和相关管理规定的行为。

如有违反，本人愿接受项目管理机构和相关部门做出的各项处理决定，包括但不限于取消项目（课题）承担资格，追回项目（课题）经费，向社会通报违规情况，取消一定期限国家科技计划项目申报资格，记入科研诚信严重失信行为数据库以及接受相应的党纪政纪处理等。

签字：[illegible]

日期：2021.8.23

课题负责人诚信承诺书：朱国行

国家科技计划项目申报诚信承诺书

（申请人部分）

本人根据国家重点研发计划项目申报指南的要求自愿提交项目（课题）申报书，**在此郑重承诺**：严格遵守《关于进一步加强科研诚信建设的若干意见》、《关于进一步弘扬科学家精神 加强作风和学风建设的意见》、《科技部 自然科学基金委关于进一步压实国家科技计划（专项、基金等）任务承担单位科研作风学风和科研诚信主体责任的通知》、《科学技术活动评审工作中请托行为处理规定（试行）》等有关规定，杜绝《科学技术活动违规行为处理暂行规定》所列违规行为，所申报材料和相关内容真实有效，不存在违背科研诚信要求的行为；已按要求落实了科研作风学风和科研诚信主体责任；不以任何形式实施请托行为，申报材料符合《中华人民共和国保守国家秘密法》和《科学技术保密规定》等相关法律法规；在参与国家科技计划项目申报、评审和实施全过程中，恪守职业规范和科学道德，遵守评审规则和工作纪律，杜绝以下行为：

（一）抄袭、剽窃他人科研成果或者伪造、篡改研究数据、研究结论；

（二）购买、代写、代投论文，虚构同行评议专家及评议意见；

（三）违反论文署名规范，擅自标注或虚假标注获得科技计划等资助；

（四）违反科研伦理规范；

（五）弄虚作假，骗取科技计划项目、科研经费以及奖励、荣誉等；

（六）在正式申报书中以高指标通过评审，在任务书签订时故意篡改降低任务书中相应指标；

（七）以任何形式探听尚未公布的评审专家名单及其他评审过程中的保密信息；

（八）本人或委托他人通过各种方式及各种途径联系有关专家进行请托、游说，违规到评审会议驻地游说评审专家和工作人员、询问评审或尚未正式向社会公布的信息等干扰评审或可能影响评审公正性的活动；

（九）向评审工作人员、评审专家等提供任何形式的礼品、礼金、有价证券、支付凭证、商业预付卡、电子红包，或提供宴请、旅游、娱乐健身等任何可能影响评审公正性的活动；

（十）其它违反财经纪律和相关管理规定的行为。

如有违反，本人愿接受项目管理机构和相关部门做出的各项处理决定，包括但不限于取消项目（课题）承担资格，追回项目（课题）经费，向社会通报违规情况，取消一定期限国家科技计划项目申报资格，记入科研诚信严重失信行为数据库以及接受相应的党纪政纪处理等。

签字：[illegible]

日期：8.23

国家科技计划项目申报诚信承诺书

（申请人部分）

本人根据国家重点研发计划项目申报指南的要求自愿提交项目（课题）申报书，**在此郑重承诺：**严格遵守《关于进一步加强科研诚信建设的若干意见》、《关于进一步弘扬科学家精神 加强作风和学风建设的意见》、《科技部 自然科学基金委关于进一步压实国家科技计划（专项、基金等）任务承担单位科研作风学风和科研诚信主体责任的通知》、《科学技术活动评审工作中请托行为处理规定（试行）》等有关规定，杜绝《科学技术活动违规行为处理暂行规定》所列违规行为，所申报材料和相关内容真实有效，不存在违背科研诚信要求的行为；已按要求落实了科研作风学风和科研诚信主体责任；不以任何形式实施请托行为，申报材料符合《中华人民共和国保守国家秘密法》和《科学技术保密规定》等相关法律法规；在参与国家科技计划项目申报、评审和实施全过程中，恪守职业规范和科学道德，遵守评审规则和工作纪律，杜绝以下行为：

（一）抄袭、剽窃他人科研成果或者伪造、篡改研究数据、研究结论；

（二）购买、代写、代投论文，虚构同行评议专家及评议意见；

（三）违反论文署名规范，擅自标注或虚假标注获得科技计划等资助；

（四）违反科研伦理规范；

（五）弄虚作假，骗取科技计划项目、科研经费以及奖励、荣誉等；

（六）在正式申报书中以高指标通过评审，在任务书签订时故意篡改降低任务书中相应指标；

（七）以任何形式探听尚未公布的评审专家名单及其他评审过程中的保密信息；

（八）本人或委托他人通过各种方式及各种途径联系有关专家进行请托、游说，违规到评审会议驻地游说评审专家和工作人员、询问评审或尚未正式向社会公布的信息等干扰评审或可能影响评审公正性的活动；

（九）向评审工作人员、评审专家等提供任何形式的礼品、礼金、有价证券、支付凭证、商业预付卡、电子红包，或提供宴请、旅游、娱乐健身等任何可能影响评审公正性的活动；

（十）其它违反财经纪律和相关管理规定的行为。

如有违反，本人愿接受项目管理机构和相关部门做出的各项处理决定，包括但不限于取消项目（课题）承担资格，追回项目（课题）经费，向社会通报违规情况，取消一定期限国家科技计划项目申报资格，记入科研诚信严重失信行为数据库以及接受相应的党纪政纪处理等。

签字：孔祥增

日期：2021.8.21

国家科技计划项目申报诚信承诺书

（申报单位部分）

本单位依据国家科技计划项目指南的任务需求，严格履行法人负责制，自愿提交申报书，**在此郑重承诺**：本单位已就所申报材料内容的真实性和完整性进行审核，不存在违背《关于进一步加强科研诚信建设的若干意见》、《关于进一步弘扬科学家精神 加强作风和学风建设的意见》、《科学技术活动违规行为处理暂行规定》、《科技部 自然科学基金委关于进一步压实国家科技计划（专项、基金等）任务承担单位科研作风学风和科研诚信主体责任的通知》、《科学技术活动评审工作中请托行为处理规定（试行）》等有关规定和其它科研诚信要求的行为，已按要求落实了科研作风学风和科研诚信主体责任；不以任何形式实施请托行为，申报材料符合《中华人民共和国保守国家秘密法》和《科学技术保密规定》等相关法律法规，在参与项目申报和评审活动全过程中，遵守有关评审规则和工作纪律，杜绝以下行为：

（一）采取贿赂或变相贿赂、造假、剽窃、故意重复申报等不正当手段获取科技计划项目承担资格；

（二）以任何形式探听未公开的评审专家名单及其他评审过程中的保密信息；

（三）组织或协助项目团队向评审工作人员、评审专家等提供任何形式的礼品、礼金、有价证券、支付凭证、商业预付卡、电子红包等；宴请评审组织者、评审专家，或向评审组织者、评审专家提供旅游、娱乐健身等可能影响评审公正性的活动；

（四）包庇、纵容项目团队虚假申报项目，甚至骗取国家科技计

划项目；

（五）包庇、纵容项目团队，甚至帮助项目团队采取“打招呼”等方式，影响评审公正；

（六）在正式申报书中以高指标通过评审，在任务书签订时故意篡改降低任务书中相应指标；

（七）其它违反财经纪律和相关管理规定的行为。

如有违反，本单位愿接受项目管理机构和相关部门做出的各项处理决定，包括但不限于停拨或核减经费，追回项目（课题）经费，取消一定期限国家科技计划项目申报资格，记入科研诚信严重失信行为数据库以及主要负责人接受相应党纪政纪处理等。

申报单位签章：

日期：2021.8.23

国家科技计划项目申报诚信承诺书

（申报单位部分）

本单位依据国家科技计划项目指南的任务需求，严格履行法人负责制，自愿提交申报书，**在此郑重承诺**：本单位已就所申报材料内容的真实性和完整性进行审核，不存在违背《关于进一步加强科研诚信建设的若干意见》、《关于进一步弘扬科学家精神 加强作风和学风建设的意见》、《科学技术活动违规行为处理暂行规定》、《科技部 自然科学基金委关于进一步压实国家科技计划（专项、基金等）任务承担单位科研作风学风和科研诚信主体责任的通知》、《科学技术活动评审工作中请托行为处理规定（试行）》等有关规定和其它科研诚信要求的行为，已按要求落实了科研作风学风和科研诚信主体责任；不以任何形式实施请托行为，申报材料符合《中华人民共和国保守国家秘密法》和《科学技术保密规定》等相关法律法规，在参与项目申报和评审活动全过程中，遵守有关评审规则和工作纪律，杜绝以下行为：

（一）采取贿赂或变相贿赂、造假、剽窃、故意重复申报等不正当手段获取科技计划项目承担资格；

（二）以任何形式探听未公开的评审专家名单及其他评审过程中的保密信息；

（三）组织或协助项目团队向评审工作人员、评审专家等提供任何形式的礼品、礼金、有价证券、支付凭证、商业预付卡、电子红包等；宴请评审组织者、评审专家，或向评审组织者、评审专家提供旅游、娱乐健身等可能影响评审公正性的活动；

（四）包庇、纵容项目团队虚假申报项目，甚至骗取国家科技计

划项目；

（五）包庇、纵容项目团队，甚至帮助项目团队采取“打招呼”等方式，影响评审公正；

（六）在正式申报书中以高指标通过评审，在任务书签订时故意篡改降低任务书中相应指标；

（七）其它违反财经纪律和相关管理规定的行为。

如有违反，本单位愿接受项目管理机构和相关部门做出的各项处理决定，包括但不限于停拨或核减经费，追回项目（课题）经费，取消一定期限国家科技计划项目申报资格，记入科研诚信严重失信行为数据库以及主要负责人接受相应党纪政纪处理等。

申报单位签章：复旦大学附属华山医院

日期：2021年8月23日

国家科技计划项目申报诚信承诺书

（申报单位部分）

本单位依据国家科技计划项目指南的任务需求，严格履行法人负责制，自愿提交申报书，**在此郑重承诺：**本单位已就所申报材料内容的真实性和完整性进行审核，不存在违背《关于进一步加强科研诚信建设的若干意见》、《关于进一步弘扬科学家精神 加强作风和学风建设的意见》、《科学技术活动违规行为处理暂行规定》、《科技部 自然科学基金委关于进一步压实国家科技计划（专项、基金等）任务承担单位科研作风学风和科研诚信主体责任的通知》、《科学技术活动评审工作中请托行为处理规定（试行）》等有关规定和其它科研诚信要求的行为，已按要求落实了科研作风学风和科研诚信主体责任；不以任何形式实施请托行为，申报材料符合《中华人民共和国保守国家秘密法》和《科学技术保密规定》等相关法律法规，在参与项目申报和评审活动全过程中，遵守有关评审规则和工作纪律，杜绝以下行为：

（一）采取贿赂或变相贿赂、造假、剽窃、故意重复申报等不正当手段获取科技计划项目承担资格；

（二）以任何形式探听未公开的评审专家名单及其他评审过程中的保密信息；

（三）组织或协助项目团队向评审工作人员、评审专家等提供任何形式的礼品、礼金、有价证券、支付凭证、商业预付卡、电子红包等；宴请评审组织者、评审专家，或向评审组织者、评审专家提供旅游、娱乐健身等可能影响评审公正性的活动；

（四）包庇、纵容项目团队虚假申报项目，甚至骗取国家科技计

划项目；

（五）包庇、纵容项目团队，甚至帮助项目团队采取"打招呼"等方式，影响评审公正；

（六）在正式申报书中以高指标通过评审，在任务书签订时故意篡改降低任务书中相应指标；

（七）其它违反财经纪律和相关管理规定的行为。

如有违反，本单位愿接受项目管理机构和相关部门做出的各项处理决定，包括但不限于停拨或核减经费，追回项目（课题）经费，取消一定期限国家科技计划项目申报资格，记入科研诚信严重失信行为数据库以及主要负责人接受相应党纪政纪处理等。

申报单位签章：厦门隆创思科技有限公司 3502061004447

日期： 2021.8.21

任务书签署

甲乙双方根据《国务院印发关于深化中央财政科技计划（专项、基金）管理改革方案的通知》（国发〔2014〕64 号）、《国务院关于优化科研管理提升科研绩效若干措施的通知》（国发〔2018〕25 号）、《国务院办公厅关于改革完善中央财政科研经费管理的若干意见》（国办发〔2021〕32 号）、《科技部 财政部关于印发<国家重点研发计划管理暂行办法>的通知》（国科发资〔2017〕152 号）、《财政部 科技部关于印发<国家重点研发计划资金管理办法>的通知》（财教〔2021〕178 号）、《科学技术活动违规行为处理暂行规定》（科学技术部令第 19 号）、《科技部财政部关于印发<中央财政科技计划（专项、基金等）监督工作暂行规定>的通知》（国科发政〔2015〕471 号）、《科技部 自然科学基金委关于进一步压实国家科技计划（专项、基金等）任务承担单位科研作风学风和科研诚信主体责任的通知》(国科发监〔2020〕203 号)等有关文件规定，以及有关法律、政策和管理要求，依据项目立项通知，签署本任务书。

同时，本单位和项目负责人**郑重承诺**：对本项目所有成果产出（包括但不限于新产品、新技术、标准、论文、专利等）的真实性、与项目的关联性等负责，将按要求落实科研作风学风和科研诚信主体责任；项目经费全部用于与本项目研究工作相关的支出，不截留、挪用、侵占，不用于与科学研究无关的支出；接受并积极配合相关部门的监督检查。如有违反，本单位和项目负责人以及相关成果产出者愿接受项目管理专业机构和相关部门做出的各项处理决定，包括但不限于终止项目执行、追回项目（课题）经费，取消一定期限国家科技计划项目申报资格，记入科研诚信严重失信行为数据库以及主要负责人接受相应党纪政纪处理等。

项目牵头承担单位（甲方）：

法定代表人签字（签章）：

（公章）

2021年12月20日

项目负责人签字（签章）：

2021年12月20日

课题承担单位（乙方）：

法定代表人签字（签章）：

（公章）

年 月

课题负责人签字（签章）：

2021 年12月20日
